# The HAPSTR2 retrogene buffers stress signaling and resilience in mammals

**DOI:** 10.1038/s41467-022-35697-1

**Published:** 2023-01-11

**Authors:** David R. Amici, Harun Cingoz, Milad J. Alasady, Sammy Alhayek, Claire M. Phoumyvong, Nidhi Sahni, S. Stephen Yi, Marc L. Mendillo

**Affiliations:** 1 Dept. of Biochemistry and Molecular Genetics, Northwestern University Feinberg School of Medicine, Chicago, IL 60610 USA; 2 Simpson Querrey Center for Epigenetics, Northwestern University Feinberg School of Medicine, Chicago, IL 60610 USA; 3 Robert H. Lurie Comprehensive Cancer Center, Northwestern University Feinberg School of Medicine, Chicago, IL 60610 USA; 4 Department of Epigenetics and Molecular Carcinogenesis, and Department of Bioinformatics and Computational Biology, The University of Texas MD Anderson Cancer Center, Houston, TX 77030 USA; 5 Quantitative and Computational Biosciences Program, Baylor College of Medicine, Houston, TX 77030 USA; 6 Livestrong Cancer Institutes, Department of Oncology, and Department of Biomedical Engineering, The University of Texas at Austin, Austin, TX 78712 USA; 7 Interdisciplinary Life Sciences Graduate Programs (ILSGP), and Oden Institute for Computational Engineering and Sciences (ICES), The University of Texas at Austin, Austin, TX 78712 USA

**Keywords:** Stress signalling, Evolutionary genetics

## Abstract

We recently identified HAPSTR1 (C16orf72) as a key component in a novel pathway which regulates the cellular response to molecular stressors, such as DNA damage, nutrient scarcity, and protein misfolding. Here, we identify a functional paralog to HAPSTR1: HAPSTR2. *HAPSTR2* formed early in mammalian evolution, via genomic integration of a reverse transcribed *HAPSTR1* transcript, and has since been preserved under purifying selection. HAPSTR2, expressed primarily in neural and germline tissues and a subset of cancers, retains established biochemical features of HAPSTR1 to achieve two functions. In normal physiology, HAPSTR2 directly interacts with HAPSTR1, markedly augmenting HAPSTR1 protein stability in a manner independent from HAPSTR1’s canonical E3 ligase, HUWE1. Alternatively, in the context of HAPSTR1 loss, HAPSTR2 expression is sufficient to buffer stress signaling and resilience. Thus, we discover a mammalian retrogene which safeguards fitness.

## Abstract (web summary)

The recently described protein HAPSTR1 governs cellular stress resilience. Here, the authors discover a mammalian HAPSTR1 paralog, called HAPSTR2, which formed via retro-transposition and operates to augment and buffer cellular stress signaling.

## Introduction

All living cells retain the ability to adapt to molecular stressors, such as those posed by changes in external environment (e.g., nutrient availability) or failures of intrinsic quality control processes (e.g., protein folding). This adaptability stems from a network of interrelated stress response signaling pathways^1^.

We recently used CRISPR screening data from cancer cell lines to identify factors that play roles in multiple stress response signaling pathways^2,3^. This analysis led us to identify HAPSTR1 (formerly: C16orf72), which despite conservation through yeast and certain plants, had no known biochemical function. We validated that HAPSTR1 broadly regulates stress signaling, in turn controlling cellular and organismal resilience^2^. Multiple other groups have also independently identified roles for HAPSTR1 in cellular stress response processes using genome-scale screens and subsequent targeted validation^4–6^.

The HAPSTR1 protein exists in mammalian cells as two isoforms, a long and a short isoform^2^, and contains two known domains: an HBO (**H**UWE1-**b**inding and HAPSTR1 **o**ligomerization) domain and a nuclear localization signal (NLS)^2^. In a seeming paradox, HUWE1—a pleiotropic ubiquitin ligase that marks HAPSTR1 for ubiquitin-dependent proteolysis—appears to be required for HAPSTR1 to regulate stress signaling^2^. However, other components of the HAPSTR1 pathway remain undetermined.

Gene duplication events provide the material from which to develop proteins with new, specialized, or supportive functions^7^. One mechanism of gene duplication is retro-transposition (retroposition), whereby a mature mRNA is reverse transcribed and integrated into the genome^8^. Classically, retrocopies lack introns and their parental gene’s promoter, and as a result, become inactive pseudogenes. However, in rare cases, retrogenes gain the ability to be transcribed and take on a function conserved throughout evolution^7,8^.

Here, we discover a paralog for HAPSTR1—HAPSTR2—which emerged through a retroposition event early in mammalian evolution. HAPSTR2 is expressed in a tissue-selective manner, where it functions dually to stabilize HAPSTR1 during normal circumstances and to preserve stress signaling when HAPSTR1 is compromised. We thus identify a novel protein-coding retrogene that buffers a conserved stress response pathway in mammals.

## Results

### A HAPSTR1 retrogene on the mammalian X chromosome

**Fig. 1:**
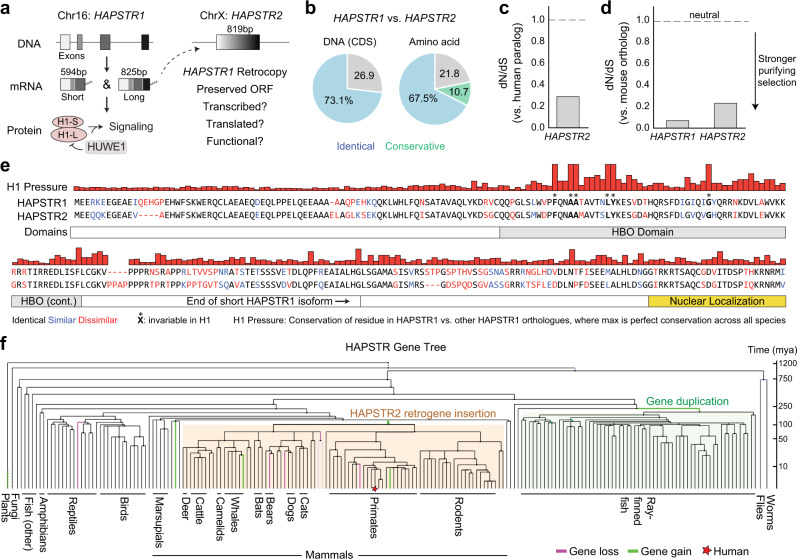
A mammalian *HAPSTR2* retrogene under purifying selection. **a** Schematic illustrating structure and known function of the *HAPSTR1* (H1) gene in comparison with an unstudied retrocopy on the X chromosome. **b** Sequence identity comparisons of *HAPSTR* genes and proteins. CDS, coding sequence. Amino acid substitutions were denoted conservative if biochemical features were preserved as determined by the BLOSUM62 matrix. **c**, **d** Purifying selection acts on *HAPSTR2* as evidenced by comparison of non-synonymous to synonymous mutation ratio (dN/dS) in human *HAPSTR2* vs. human *HAPSTR1* (**c**) or human *HAPSTR* genes vs. their murine orthologs (**d**). Note that dN/dS of 1 indicates complete neutrality and a value less than 0.5 is a more conservative threshold for functionality when comparing parental gene/retrogene pairs^24,44^. **e** Comparison of HAPSTR1 with the predicted HAPSTR2 protein. Differences, domains, and degree of conservation for each residue among HAPSTR1 orthologs highlighted; note legend at bottom. Invariable indicates perfect conservation in all HAPSTR1 species. **f** Tracking *HAPSTR* gene gain and loss events throughout evolution; note legend in bottom right and scale on right.

In searching for genes that share sequence similarity with *HAPSTR1* (Chr16), we identified a genomic region (*RP11-364B14.3/LOC389895*) on the human X chromosome which contains an open reading frame highly similar to the *HAPSTR1* coding sequence (100% coverage, 73% nucleotide identity, 78% codon similarity; Fig. 1a, b). Notably, this copy of *HAPSTR1*—henceforth called *HAPSTR2*—lacked any introns, suggesting an evolutionary origin as a retro-transposed *HAPSTR1* transcript^7,8^. Further indicating the origin of *HAPSTR2* as a retrocopy, we observed full coverage of the *HAPSTR1* coding sequence (CDS) and remnants of a poly(A) tail with target site duplications (Supplementary Fig. 1). Recently, the Human and Vertebrate Analysis and Annotation (HAVANA) project also manually annotated *HAPSTR2* as a putative protein-coding retrogene (Wellcome Sanger Institute).

Inactive retrocopies face no selection pressure, accumulating mutations rapidly which often disrupt the original reading frame^8^. However, *HAPSTR2’s* sequence similarity to *HAPSTR1* and reading frame preservation suggested that HAPSTR2 encodes a functional product (Fig. 1b). Comparison of the synonymous (silent) and non-synonymous mutation rates for *HAPSTR2* demonstrated that *HAPSTR2* is under negative (purifying) selection pressure, further implying that *HAPSTR2* is beneficial for organismal fitness (Fig. 1c, d)^9^.

We next compared the HAPSTR protein amino acid sequences. Notably, each HAPSTR1 residue perfectly conserved throughout HAPSTR1’s evolution—a group with critical functions for oligomerization and HUWE1-binding^2^, comprising F90, AA93-94, LY100-101, and G119—were also conserved in HAPSTR2 (Fig. 1e, Supplementary Fig. 2a). HAPSTR2 protein structure predictions with AlphaFold2^10,11^ were highly similar to HAPSTR1 (Supplementary Fig. 2b).

Given the presence of *HAPSTR1* orthologs throughout metazoans as well as certain fungi and plants, we undertook an evolutionary analysis to identify when *HAPSTR2* emerged. Focusing on early events in metazoan evolution, we found two distinct duplication events for *HAPSTR1*. The first, occurring ~280 million years ago, produced a second copy of *HAPSTR1* in ray-finned fish (e.g., zebrafish; Fig. 1f, Supplementary Fig. 2c). The presence of introns suggested that this was a duplication of the *HAPSTR1* chromosomal segment (Supplementary Fig. 2c). The second, occurring ~180 million years ago, occurred early in mammalian evolution, just after the split of eutherian/placental mammals from marsupials (Fig. 1f). This second event yielded the mammalian *HAPSTR2* retrogene, characterized by the lack of introns and localization to the X chromosome (Fig. 1f, Supplementary Fig. 2d). Taken together, our data indicate that a retroposition event early in mammalian evolution produced a copy of *HAPSTR1* which was retained in the genome under purifying selection pressure.

### Expression of *HAPSTR2* in a tissue-restricted fashion

**Fig. 2:**
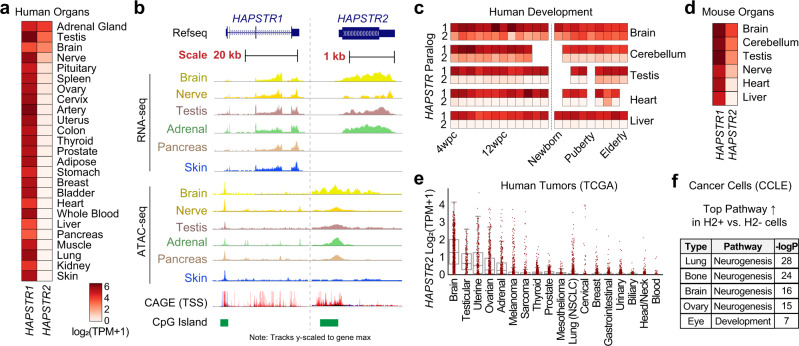
Tissue-restricted expression of mammalian *HAPSTR2*. **a** Transcript abundance of *HAPSTR1* and *HAPSTR2* in the indicated human tissues; GTEx RNA-seq data. TPM, transcripts per million. Same heatmap scale across other subpanels. **b** Gene expression, chromatin accessibility, transcription start sites, and CpG islands at human *HAPSTR1* and *HAPSTR2* gene loci; GTEx, ENCODE, and FANTOM5 data. Note that some intron 3 reads for *HAPSTR1* correspond to an expressed lncRNA (ENSG00000263244). kb, kilobases. Note the relative track scaling; refer to 2a for expression estimates. **c** Expression of *HAPSTR* genes throughout the lifespan in different organs^13^. **d** Expression of *HAPSTR* genes in murine tissues; MGI data. **e** Expression of *HAPSTR2* in tumors; TCGA data, *n* = 10535. Box is median and lower/upper quartile with whiskers double the interquartile range. **f** Top-ranked ontological enrichment for genes upregulated in *HAPSTR2*-expressing vs. non-expressing cancer cell lines within the indicated lineage; CCLE RNA-seq data.

To assess the extent to which *HAPSTR2* is transcribed, we analyzed RNA-sequencing (RNA-seq) data from a collection of diverse tissues^12^. We found that *HAPSTR2* is indeed expressed, but only in a subset of tissues: the testis—the most common site for retrogene expression due to a permissive chromatin environment^7^—but also neural tissues such as the brain, peripheral nerve, and adrenal gland (Fig. 2a, b). This is in contrast with *HAPSTR1*, which is ubiquitous across tissues (Fig. 2a, b). The divergence between *HAPSTR1* and *HAPSTR2* transcript abundance in certain tissues indicates that *HAPSTR2* RNA-seq reads do not reflect misalignment of *HAPSTR1* reads. Further demonstrating the validity of RNA-seq data to specifically quantify *HAPSTR2* vs. *HAPSTR1*, we validated gene expression predictions for each paralog using paralog-specific RT-qPCR primers, exogenous overexpression, and knockdown experiments (Supplementary Fig. 3a–c).

To better understand the landscape of *HAPSTR2* expression, we queried other resource transcription datasets. Gene expression profiling of human organs throughout the lifespan^13^ confirmed the tissue-restricted nature of *HAPSTR2* expression and additionally suggested that *HAPSTR2* levels are regulated dynamically during development; for example, higher *HAPSTR2* in pre- vs. post-natal cerebellum and in post- vs. pre-pubertal testis (Fig. 2c). Murine tissues demonstrated the same pattern of *HAPSTR* paralog expression as human tissues (Fig. 2d)^14^, indicating that the tissue distribution of *HAPSTR2* relative to *HAPSTR1* is conserved in other mammals.

Given the apparent importance of *HAPSTR1* in cancer^2,6^, we also investigated *HAPSTR2* expression in tumors and cancer cell lines. Consistent with organ expression patterns, tumors derived from brain, adrenal, and germline tissues tended to have the highest expression of *HAPSTR2* (Fig. 2e). Tumor *HAPSTR2* was typically low relative to *HAPSTR1*, but a notable subset of tumors expressed comparable amounts of both paralogs (Supplementary Fig. 3d). Cancer cell lines similarly recapitulated tissue biases in *HAPSTR2* expression (Supplementary Fig. 3e). Yet, we were intrigued to observe *HAPSTR2* expression in a subset of cancers from tissues which do not normally express *HAPSTR2* (Supplementary Fig. 3e). Among well-characterized cancer cell lines, this was most notable in lung and bone cells. To better understand the contexts in which cancer cells express *HAPSTR2*, we investigated genes differentially expressed in *HAPSTR2*-positive vs. *HAPSTR2*-negative cancer cell lines from a given lineage. *HAPSTR2*-expressing subsets of bone and lung cancer were characterized by striking overexpression of genes involved in neural linage determination (Fig. 2f). Neurogenesis genes also delineated *HAPSTR2*-high vs. -low brain and ovarian cancer cell lines (Fig. 2f), altogether consistent with our observations that neural lineage factors likely drive *HAPSTR2* expression. Notably, we found no link between *HAPSTR2* transcript abundance and *HAPSTR1* expression, *HAPSTR1* mutations, or *HAPSTR2* copy number (Supplementary Fig. 3f, g).

Considering the origin of *HAPSTR2* as a retrocopy, we next investigated the evolutionary mechanism which facilitated *HAPSTR2* expression. Retrocopies gain the ability to be expressed via one of three mechanisms: inheritance of the parental promoter (when the retroposed mRNA stems from an upstream transcription start site (TSS)), “piggybacking” the promoter of a nearby gene, or evolution of novel regulatory elements^7^. We identified the *HAPSTR2* promoter from transcription start site mapping (CAGE) data (Fig. 2b)^15^. *HAPSTR2’s* promoter did not align with *HAPSTR1’s* promoter (Supplementary Fig. 3h) and there are no neighboring genes to *HAPSTR2* within 50 kb, ruling out the inheritance of the parental promoter and piggybacking, respectively. Thus, *HAPSTR2* was either inserted downstream of a proto-regulatory region, with subsequent evolution towards promoter function, or a promoter developed de novo by base substitutions. CpG islands—which can act as rudimentary promoters even when not associated with transcripts and are enriched near expressed retrogenes vs. inactive retrogenes—are thought to be common proto-regulatory regions facilitating initial retrogene expression^7,16^. We observed a CpG island near *HAPSTR2’s* TSS which may have represented the initial mechanism of *HAPSTR2* retrocopy expression (Fig. 2b). Together, our data suggest that *HAPSTR2* inserted into the genome absent its parental gene’s promoter, where subsequent evolutionary refinement of a proto-promoter resulted in transcription in neural and germline tissues and a subset of cancers.

### *HAPSTR2* encodes a protein that retains the biochemical features of HAPSTR1

Transcription of a retrogene does not always indicate that a functional protein is produced. We first confirmed that *HAPSTR2* mRNA encodes a stable protein by introducing *HAPSTR2* cDNA to 293T cells (Supplementary Fig. 4a). We also produced recombinant maltose-binding protein (MBP)-tagged HAPSTR2 to facilitate in vitro assays (Supplementary Fig. 4b). Of note, we tested the ability of our HAPSTR1 antibody to detect the HAPSTR2 protein. Despite 78% amino acid identity (89% similarity) to the HAPSTR1 protein in the region bound by our antibody (residues 1-45), HAPSTR2 was ~1000-fold less efficiently detected by the HAPSTR1 antibody in immunoblots (Supplementary Fig. 4c, d).

**Fig. 3:**
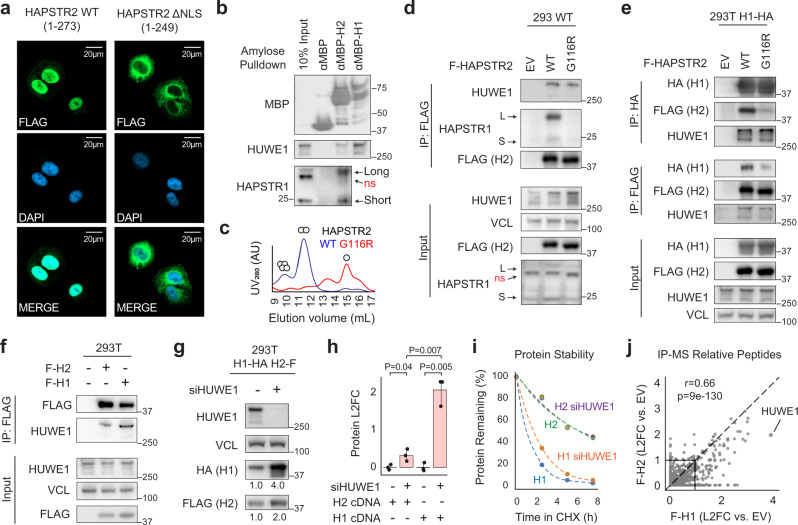
HAPSTR2 retains biochemical features of HAPSTR1 but less avidly binds HUWE1. **a** HAPSTR2 localizes primarily to the nucleus in a manner dependent on a conserved C-terminal nuclear localization signal (NLS; residues 250–273). Field representative of three independent experiments. **b** Isolation of prey proteins from 293T cell lysates using recombinant MBP, MBP-HAPSTR2 (H2), or MBP-HAPSTR1 (H1). Note that HAPSTR1 on immunoblot appears as a long and short isoform with a non-specific band in between, and that HAPSTR2 co-purifies both isoforms but not the non-specific protein^2^. Representative of *N* = 3. **c** Analytical size exclusion chromatography of wild-type (WT) or the oligomerization-impaired (G116R) mutant recombinant protein, with stoichiometry estimates indicated. Representative of *N* = 3. **d**–**e** Co-immunoprecipitations (co-IPs) isolating WT or mutant FLAG-tagged HAPSTR2 from otherwise parental (**d**) or HAPSTR1-HA overexpressing (**e**) 293T cells. **f** FLAG-HAPSTR2 (F-H2) co-IPs endogenous HUWE1 less efficiently than does FLAG-HAPSTR1 (F-H1). Representative of *N* = 3. **g**–**h** HUWE1 depletion destabilizes HAPSTR1 more substantially than HAPSTR2 in cells co-expressing both HAPSTR paralogs with different tags (**g**) or expressing each HAPSTR paralog in isolation (**h**). Two-tailed *t* test. Both experiments *N* = 3. Mean ± 95% confidence interval. cDNA, complementary DNA. L2FC, log_2_-fold-change. **i** Turnover rates of co-expressed HAPSTR1 (H1) and HAPSTR2 (H2) after treatment with 40 µM cycloheximide (CHX) to stop translation. Cells were pre-treated for 48 hours with either HUWE1 siRNA (siHUWE1) or a non-targeting control siRNA. *N* = 3. **j** Mass spectrometry (MS) analysis of HAPSTR protein co-IPs. Mean of two independent co-IP/MS experiments per protein/control. See Supplementary Data 1. EV, empty vector.

Next, we tested whether HAPSTR2 retains the established biochemical functions of the HAPSTR1 protein: the ability to localize to the nucleus, oligomerize, and bind the E3 ligase HUWE1^2^. We first tested localization, revealing that HAPSTR2—like HAPSTR1^2^—localizes to the nucleus in a manner dependent on a conserved C-terminal NLS (Fig. 3a).

We subsequently tested HAPSTR2’s protein binding capacities. Using recombinant MBP-HAPSTR1, MBP-HAPSTR2, or MBP alone as bait, we isolated prey proteins from a whole cell lysate, finding that HAPSTR2 co-purified endogenous HAPSTR1 (both isoforms) as well as HUWE1 (Fig. 3b).

The in vitro interaction of HAPSTR2 with cellular HAPSTR1 led us to further query HAPSTR2’s ability to homo- and hetero-oligomerize. Analytical size exclusion chromatography of recombinant HAPSTR2 indicated that, like HAPSTR1^2^, HAPSTR2 homo-oligomerizes in a manner which requires an HBO domain glycine (G116; G119 in HAPSTR1) perfectly conserved in all HAPSTR proteins throughout evolution (Fig. 3c, Supplementary Fig. 2a). Co-immunoprecipitations (co-IPs) in cells demonstrated that HAPSTR2 hetero-oligomerizes with HAPSTR1 using the same HBO domain interface (Fig. 3d, e). Notably, the G116R (oligomerization-deficient) HAPSTR2 mutant still bound HUWE1, indicating that HAPSTR2 does not require HAPSTR1 to bind HUWE1 (Fig. 3d, e).

Notably, in our parallel in vitro pulldowns, less HUWE1 was co-purified by HAPSTR2 as compared with HAPSTR1, suggesting a weaker binding affinity (Fig. 3b). Co-IPs in vivo also demonstrated a moderately reduced ability for HAPSTR2 to co-purify HUWE1 (Fig. 3f). Testing the functional consequence of this observation, we found that HAPSTR2 was much less strongly destabilized by HUWE1 as compared with HAPSTR1 (Fig. 3g, h). We further determined the kinetics of HAPSTR protein turnover in cells expressing both paralogs with different tags. This revealed that HAPSTR2 is ~4-fold more stable than HAPSTR1 in a manner partially explained by differential HUWE1 regulation (Fig. 3i, Supplementary Fig. 4e).

Finally, we performed unbiased immunoprecipitation coupled with mass spectrometry (IP-MS) to identify any other major differences in the protein interactome of HAPSTR1 and HAPSTR2 (Fig. 3j, Supplementary Data 1). HUWE1 was by far the strongest interacting partner for HAPSTR1, consistent with prior experiments^2,5^. The HUWE1 interaction was weaker for HAPSTR2, corroborating our biochemical experiments. Regardless, the overall interactomes between HAPSTR1 and HAPSTR2 were very similar (*r* = 0.66; *p* = 9e-130). Altogether, our data indicate that HAPSTR2 retains the biochemical features of HAPSTR1 but has diminished ability to bind and be regulated by HUWE1.

### HAPSTR2 stabilizes HAPSTR1 through a direct physical interaction

**Fig. 4:**
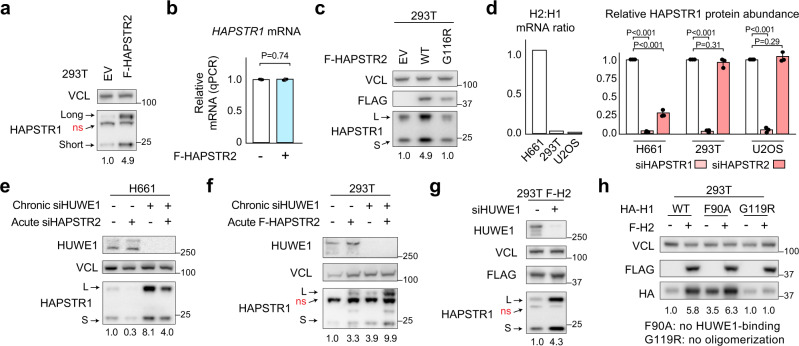
HAPSTR2 stabilizes HAPSTR1 through a direct physical interaction. **a**, **b** Expression of FLAG-tagged (F)-HAPSTR2 increases endogenous HAPSTR1 protein abundance in 293T cells (**a**) without affecting HAPSTR1 transcript abundance (**b**). Representative of *N* = 5 (**a**/**b**). Note that HAPSTR1 on immunoblot appears as a long (L) and short (S) isoform with a non-specific (ns) band in between^2^, and that HAPSTR2 augments both isoforms but not the non-specific protein (**a**). Mean ± 95% confidence interval (CI), two-tailed *t* test (**b**). **c** Hetero-oligomerization is required for HAPSTR2-mediated HAPSTR1 stabilization. *N* = 5. **d** Effect of HAPSTR2 knockdown on endogenous HAPSTR1 protein abundance in cells which express HAPSTR2 (H661) or do not express HAPSTR2 (293 T, U2OS). Two-tailed *t* test. *N* = 3. Mean ± 95% CI. **e** HAPSTR2 knockdown by siRNA (siHAPSTR2) in cells already lacking HUWE1 via siRNA (siHUWE1) still destabilizes HAPSTR1. *N* = 3. **f** HAPSTR2 overexpression increases endogenous HAPSTR1 abundance in a manner that does not require HUWE1. *N* = 3. **g** HUWE1 still destabilizes HAPSTR1 in the presence of HAPSTR2. *N* = 3. **h** HAPSTR1-F90A which cannot interact with HUWE1^2^ is still stabilized by HAPSTR2 overexpression, but an oligomerization interface mutant (G119R) suppresses the effect. *N* = 3.

When overexpressing HAPSTR2, we noticed a striking increase in HAPSTR1 protein abundance, affecting both HAPSTR1 isoforms without altering *HAPSTR1* mRNA abundance (Fig. 4a, b). Mutating the oligomerization interface (HAPSTR2-G116R) suppressed this effect, indicating that HAPSTR2 stabilizes the HAPSTR1 protein via direct physical interaction (Fig. 4c).

To assess whether native HAPSTR2 mediates HAPSTR1 stabilization, we performed the reciprocal experiment, knocking down endogenous HAPSTR2 in a cell line (H661) which natively expresses similar amounts of HAPSTR1 and HAPSTR2 mRNA (Fig. 4d). As compared with cell lines with minimal HAPSTR2 expression, where HAPSTR2 knockdown had no effect on HAPSTR1 abundance, HAPSTR2 knockdown in H661 cells resulted in a 69% reduction in HAPSTR1 protein (Fig. 4e, Supplementary Fig. 4f). Thus, in cells expressing HAPSTR2, HAPSTR2-mediated stabilization of HAPSTR1 plays a major role in the cellular availability of HAPSTR1.

The opposing effects of HAPSTR2 and HUWE1 on HAPSTR1 stability led us next to test whether HAPSTR2 functionally protects HAPSTR1 from HUWE1-mediated degradation. However, several orthogonal lines of evidence suggested that HAPSTR2 stabilizes HAPSTR1 independently from HUWE1. In cells chronically depleted of HUWE1, acute HAPSTR2 knockdown and overexpression still regulated HAPSTR1 protein levels (Fig. 4e, f). Furthermore, in cells stably overexpressing HAPSTR2, HUWE1 still destabilized HAPSTR1 (Fig. 4g). Finally, the HAPSTR1-F90A mutant which does not bind HUWE1^2^ was stabilized by HAPSTR2, whereas the HAPSTR1-G119R mutant which does not bind HAPSTR2 was unaffected (Fig. 4h). Thus, HAPSTR2 directly binds and stabilizes HAPSTR1 in a manner which does not require HUWE1.

### HAPSTR2 augments and safeguards HAPSTR1-dependent stress signaling

We originally identified HAPSTR1 through genome-wide analyses for factors likely to impact multiple stress response signaling pathways^2^. We subsequently found that the HAPSTR1-HUWE1 pathway regulates a broad variety of stress responses, including effects on basal and inducible expression of several stress signaling proteins like p53/TP53, p21/CDKN1A, and HO-1/HMOX1. We thus sought to investigate the functional impact of HAPSTR2 on HAPSTR1-dependent stress signaling.

**Fig. 5:**
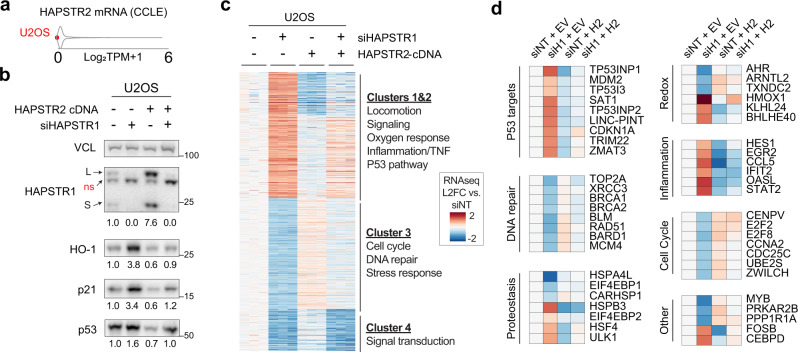
HAPSTR2 augments and buffers stress signaling. **a** Plot illustrating low (0 TPM) expression of endogenous HAPSTR2 in U2OS cells. CCLE: cancer cell line encyclopedia. **b** Expression of exogenous HAPSTR2 is sufficient to augment and buffer HAPSTR1-dependent stress signaling. Note that HAPSTR1 on immunoblot appears as a long (L) and short (S) isoform with a non-specific (ns) band in between^2^. Mean of *N* = 3 quantified. **c**, **d** RNA-sequencing of U2OS cells after HAPSTR1 depletion by siRNA (siHAPSTR1) and/or overexpression of HAPSTR2. *N* = 3. All differentially expressed genes after HAPSTR1 knockdown are shown (**c**) and selected pathways are highlighted (**d**). See Supplementary Data 2.

In cells lacking endogenous HAPSTR2 (U2OS), ectopic HAPSTR2 expression was sufficient to increase HAPSTR1 levels and enact signaling changes opposite to those caused by impairment of the HAPSTR1-HUWE1 pathway^2^ (Fig. 5a, b). This observation suggests that HAPSTR2 expression augments basal HAPSTR-HUWE1 pathway function. We then assessed whether HAPSTR2 can compensate for HAPSTR1 loss, a phenomenon called paralog buffering which is thought to safeguard organismal fitness^17–19^. Indeed, HAPSTR2 expression either fully or partially rescued the effects of HAPSTR1 loss on model signaling proteins (Fig. 5b).

To extend these findings beyond model proteins, we performed RNA-sequencing as an unbiased readout (Supplementary Data 2). As expected, HAPSTR1 depletion markedly remodeled transcriptome-wide signaling, including gene sets related to DNA damage/repair, protein homeostasis (proteostasis), and redox stress responses (Fig. 5c, d). Most alterations were either completely or partially rescued by introduction of HAPSTR2, which was also sufficient to invoke opposite changes to HAPSTR1 loss in HAPSTR1-WT cells (Fig. 5c, d, Supplementary Fig. 5a). Altogether, these data suggest that HAPSTR2 buffers HAPSTR pathway function in WT cells and can buffer HAPSTR1-dependent signaling when HAPSTR1 function is compromised.

### HAPSTR2 buffers resilience in the context of HAPSTR1 loss

**Fig. 6:**
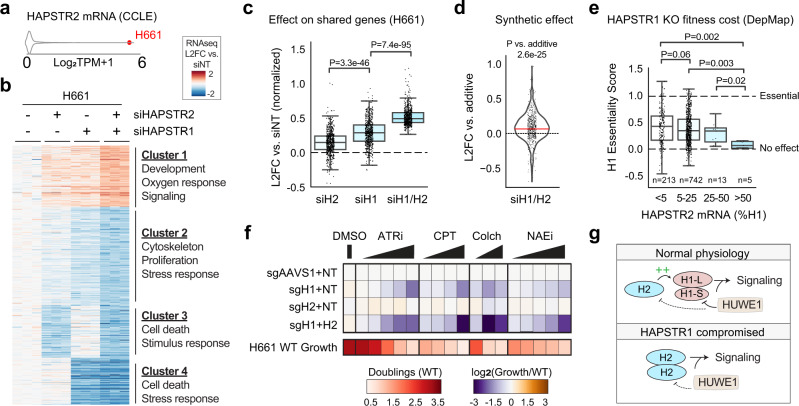
HAPSTR2 safeguards resilience. **a** Plot illustrating relatively high expression of endogenous HAPSTR2 in H661 cells. **b** RNA-sequencing of HAPSTR paralog depletion in isolation or together using siRNA; all differentially expressed genes in the double knockdown condition are shown. See Supplementary Data 3. *N* = 3. **c** Magnitude of effects on gene expression for the indicated perturbation, where each point is a gene (*n* = 589) from cluster 1 or 2. Note that the fold change for genes in Cluster 2 is multiplied by -1 to include both up- and down-regulated genes. Mann–Whitney *U*, two-tailed**. d** The observed gene expression change after double paralog knockdown, relative to the expected effect if siHAPSTR1 and siHAPSTR2 were acting independently (and thus added together). L2FC: Log-2-fold change. 589 genes, one-sample *t* test. **e** HAPSTR1 knockout (KO) via CRISPR-Cas9 markedly reduces fitness in most cancer cell lines, but less so in cell lines expressing relatively high amounts of HAPSTR2. Note that the essentiality score here is a continuous measure, where 0 is no fitness effect and 1 is the average growth defect of targeting genes considered universally required for cell growth. *N* = 973 cell lines analyzed (group breakdown in figure). Mann–Whitney *U*, one-tailed. Box is median and lower/upper quartile with whiskers 150% of the interquartile range. **f** Stress tolerance, quantified as 6-day growth relative to wild-type (WT) cells, of H661 population knockout cells created via CRISPR-Cas9 sgRNAs targeting HAPSTR paralogs, AAVS1 (safe harbor control), and/or a non-targeting (NT) sgRNA. *N* = 5. Colch, colchicine; ATRi, ATR inhibitor AZD6738; NAEi, Neddylation inhibitor MLN4924; CPT, camptothecin. **g** Schematic model of two functions for HAPSTR2 (H2), augmenting HAPSTR1 (H1) stability normally and safeguarding against total loss of HAPSTR functionality when HAPSTR1 is lost.

HAPSTR2 is expressed primarily in neural and germline tissues, as well as neural, germline, and neural-like cancers. To further test whether HAPSTR2 functionally buffers HAPSTR1 loss, we investigated neural-like lung cancer cells, H661, which natively express both HAPSTR1 and HAPSTR2 (Figs. 6a, 4d).

We first investigated the signaling consequences of depleting HAPSTR1, HAPSTR2, or both proteins simultaneously via RNA-sequencing (Supplementary Data 3). While each individual knockdown affected a small set of unique genes, potentially reflecting siRNA off-target effects, a core shared set of transcripts was regulated by knockdown of each paralog (Clusters 1&2, Fig. 6). Despite comparable basal paralog expression (Fig. 4d) and knockdown efficiency (Supplementary Fig. 5b), HAPSTR1 knockdown had stronger effects on gene expression than HAPSTR2 (Fig. 6c). Importantly, double knockdown provided a larger effect than depletion of either paralog (Fig. 6c). This effect was synthetic (Fig. 6d)—that is, greater than expected by adding the individual gene expression changes (Supplementary Fig. 5c)—suggesting functional buffering between endogenous HAPSTR proteins consistent with our complementation studies.

To investigate the functional consequences of this redundancy, we investigated whether cell lines that natively express HAPSTR2 are protected from the fitness cost of HAPSTR1 knockout. Leveraging data from the Dependency Map (DepMap) project of 1000 cell lines, we found that HAPSTR2 abundance indeed correlated with reduced reliance upon HAPSTR1 for growth (Fig. 6e). To directly test functional buffering by HAPSTR2, we knocked out *HAPSTR1*, *HAPSTR2*, or both genes using CRISPR-Cas9. Knockout of *HAPSTR2* in H661 cells—but not 293T cells, which do not meaningfully express *HAPSTR2* (Fig. 4d)—reduced HAPSTR1 protein abundance 66%, confirming functional knockout (Supplementary Fig. 5d). HAPSTR1-KO, HAPSTR2-KO, and double KO (DKO) H661 cells did not proliferate differently from WT cells in basal, non-stressed conditions (Fig. 6f). However, in the presence of stressors previously linked to HAPSTR1 or HUWE1^2,4,20^, HAPSTR1-KO cells were less fit than WT cells (Fig. 6f). HAPSTR2-KO alone had no effect on resilience to these stressors, but when combined with HAPSTR1-KO, there was a marked synthetic sick interaction (Fig. 6f, Supplementary Fig. 5c). That is, HAPSTR2 was dispensable at baseline, but required to prevent a collapse of stress tolerance when HAPSTR1 was compromised.

Altogether, our data indicate a retroposition event early in mammalian evolution created a functional, tissue-restricted HAPSTR1 paralog which operates to buffer resilience (Fig. 6g).

## Discussion

A central goal of genetics is to assign a function to all protein-coding genes. Progress towards this goal has accelerated in recent years as CRISPR-based screening studies implicate important but historically unstudied genes, such as *HAPSTR1* (formerly: *C16orf72*)^2,4,5,20^. However, common CRISPR libraries do not target genes of uncertain protein-coding capacity, like *HAPSTR2* (once annotated as the noncoding pseudogene *RP11-364B14.3*). Our evolutionary and biochemical analyses identifying the *HAPSTR2* retrogene thus both identifies a novel protein-coding gene and augments our understanding of an ancient stress resilience pathway.

How common is the mechanism by which *HAPSTR2* emerged? Retroposition of mammalian transcripts occurs nonspecifically via the LINE-1 retrotransposon machinery^21^ and has produced upwards of 10,000 retrocopies resident in the human genome^22^. Yet, only 1–2% of retrocopies in humans are both expressed and subject to purifying selection, indicating that the vast majority of retrocopies do not encode functional proteins^7,8,22^. *HAPSTR2* thus represents a relatively rare case whereby a retrocopy lacking a promoter and introns was evolutionarily resuscitated to take on a function beneficial for organismal fitness.

The X chromosome differs in many ways from autosomal chromosomes^23^ and has served as a hub for retrogene traffic throughout mammalian evolution^24^. X-to-autosome retroposition events are thought to create a substitute for genes critical for spermatogenesis, during which the X is temporarily silenced. On the other hand, the rationale for frequent autosome-to-X transfers, like we observe with *HAPSTR2*, is less clear. One implication of X linkage is “faster” evolution, since mutations will have greater penetrance in hemizygous males^23,25^. A second implication is that X-linked genes are more frequently sex-specific in expression or importance^23–25^. We found no evidence for sex-specificity in *HAPSTR2* expression, but future studies in vivo will be required to investigate sex-specific consequences for physiology.

Paralogs typically undergo some degree of functional differentiation from their parent gene^26^. What, then, differentiates HAPSTR2 from HAPSTR1? We identify two major differences: transcriptional regulation and HUWE1-binding.

Regarding transcription, while *HAPSTR1* is ubiquitous, *HAPSTR2* is largely restricted to germline and neural tissues. Evolutionarily, retrocopies are often first expressed in the testis due to a permissive chromatin environment; thereafter, retrocopies can develop a function that is advantageous to extend to other tissues^7^. This observation, considering our data indicating that *HAPSTR2’s* promoter developed from a non-specific proto-promoter, suggests that HAPSTR2’s function may be particularly advantageous in the neural context. It is thus noteworthy that genomic alterations in *HAPSTR1*^2^, *HAPSTR2* (Supplementary Data 4), and several factors predicted or demonstrated to be important for the HAPSTR pathway (e.g., HUWE1, USP7, and TRIP12)^27–35^ have been identified in individuals with neurodevelopmental disorders. Thus, HAPSTR2 may represent a valuable buffering mechanism for a pathway critical in neural tissues. Residual expression of HAPSTR2 in some neural or germline cancers (and de novo expression in neural-like subsets of other cancers) also underscores a need to consider paralog effects in future studies of HAPSTR1 in cancer.

The second differentiating factor between HAPSTR1 and HAPSTR2 concerns HUWE1-binding affinity. Our data suggest that HUWE1 more avidly binds and regulates HAPSTR1, while HAPSTR2 is relatively protected from HUWE1-mediated degradation. This difference contributes to the ~4-fold greater stability of HAPSTR2 relative to HAPSTR1, an observation with major implications for relative HAPSTR1/2 protein abundance in tissues or tumors where both paralogs are transcribed. More broadly, we reason that the weaker HAPSTR2::HUWE1 interaction enables a system whereby competing HAPSTR1 regulatory mechanisms remain independent and energy efficient. That is, tissue-specific expression of HAPSTR2 can boost HAPSTR1 protein levels without blocking normal HUWE1-HAPSTR1 dynamics, whereas fluctuations in HUWE1 activity can tune HAPSTR1 levels without turning off an alternative regulatory mechanism.

Another implication of HAPSTR2’s HUWE1-binding considers our prior data suggesting that HUWE1 is required for HAPSTR1 to control stress signaling, and that HUWE1-mediated HAPSTR1 degradation may serve as a feedback mechanism^2^. Thus, the observation that HAPSTR2 can still complex with HUWE1 independent from HAPSTR1 suggests the possibility of HAPSTR2 replacing HAPSTR1 in the HAPSTR-HUWE1 complex in contexts where HAPSTR1 is compromised. This notion of paralog buffering has been observed with many other paralogous gene pairs^17–19^ and is supported by our signaling and functional experiments. We note here a distinction between buffering of HAPSTR1 loss via constitutive expression of its paralog and genetic compensation, whereby one paralog is selectively expressed in response to the loss of the other^36–38^. Indeed, we observed no evidence that *HAPSTR2* is selectively expressed upon the loss of HAPSTR1. We propose that the additive advantage of constitutive (versus solely compensatory) expression of HAPSTR2 is to stabilize HAPSTR1 and tune stress signaling in WT cells.

In addition to the retrogene mechanism we identify in placental mammals, we note that the *HAPSTR1* gene has been duplicated several other times throughout evolution. Examples include a duplication early in the evolution of fish (Fig. 1e, Supplementary Fig. a) as well as events beyond metazoans (e.g., in several lineages of asterid plants). Thus, the buffering of HAPSTR1 function through paralog acquisition represents a strategy beneficial for the fitness of multicellular organisms throughout multiple kingdoms of life.

## Methods

### Evolutionary analyses and sequence information

Nucleotide and protein sequences for all comparisons were downloaded from Ensembl using the following IDs: *HAPSTR1*, ENSG00000182831; HAPSTR2, ENSG00000230707. Multi-sequence alignment was performed using Clustal Omega v1.2.4 as accessed at https://www.ebi.ac.uk/Tools/msa/clustalo/, with visualization of the alignment in Jalview v2.11.

For analysis of selection pressures, the non-synonymous to synonymous nucleotide substitution rates (Ka/Ks) for HAPSTR2 versus human *HAPSTR1* and mouse *HAPSTR2* were calculated using a previously defined method^9,39^ implemented at services.cbu.uib.no/tools/kaks/index_html. For alignment of the *HAPSTR2* promoter with that of *HAPSTR1* (parental gene) and *TUBB* (negative control), we aligned the 1000 bp upstream of *HAPSTR2’s* start codon with the region 5000 bp upstream of *HAPSTR1* and *TUBB* using the DNAFULL matrix implemented in SnapGene (v5.3).

For analyses of *HAPSTR1* duplications over evolutionary history, we curated a *HAPSTR1* gene tree. The *HAPSTR1/C16orf72* gene gain/loss tree was downloaded from Ensembl and then edited based on manual curation and comparisons with the PFAM DUF4588 tree. We note that, as compared with Ensembl, PFAM’s domain of unknown function DUF4588 tree—focusing just on *HAPSTR1’s* central domain—identifies more distant orthologs (i.e., fungal and plant orthologs) to *HAPSTR1*. The plant lineage is left unconnected (dashed line) to fungal and metazoan lineages in the tree as it is uncertain the extent to which plant *HAPSTR1* emerged via convergent versus divergent evolution.

### Public gene expression resources for normal tissues

Human tissue gene expression data were obtained via the GTEx project V8 (https://GTExportal.org/home/)^12^. Human *HAPSTR1* (*C16orf72*) and *HAPSTR2* (*RP11-364B14.3*) were queried. Median-grouped data by tissue were then plotted. Mouse tissue gene expression data were obtained through the Mouse Genome Informatics tool (informatics.jax.org)^14^. Mouse *HAPSTR1* (*1810013L24Rik*) and *HAPSTR2* (*Gm715*) were queried, yielding 1350 processed RNA-seq experiments with TPM values. Of these, experiments were filtered by structure for the indicated tissues (note: cerebral cortex was used for brain, sciatic nerve for nerve, and renal tubule for kidney) prior to grouping by median. Gene expression values in different organs throughout the lifespan were obtained from Cardoso-Moreira et al^13^.

Genome browser visualizations for GTEx data used data from a public session hosted on the UCSC browser (https://genome.ucsc.edu/GTEx.html). ATAC-seq data were obtained from ENCODE (https://www.encodeproject.org/) by querying the experiment search tool for ATAC-seq and *Homo sapiens* datasets. Data for tissues of interest was then visualized on the UCSC browser, and representative peaks shown. Finally, CAGE data from FANTOM^15^, hosted on the UCSC browser, were visualized.

### Phenotyping data for tumors and cancer cell lines

Tumor RNA-seq data were obtained from XenaBrowser (UCSC; https://xenabrowser.net/datapages/). The dataset chosen was the TCGA Pan-Cancer study (*n* = 10,535), version 2016-09-01, processed by UCSC TOIL RNA-seq recompute (RSEM) and yielding TPM values (dataset ID: tcga_RSEM_gene_tpm). The accompanying “sample type and primary disease” metadata file was used to map expression values to tumor types. Finally, for visualization purposes, the cancer subtypes were grouped as follows. Breast: breast invasive carcinoma.; Brain: glioblastoma multiforme, brain lower grade glioma.; Head/Neck: esophageal carcinoma, head & neck squamous cell carcinoma.; Lung (NSCLC): lung adenocarcinoma, lung squamous cell carcinoma.; Gastrointestinal: colon adenocarcinoma, stomach adenocarcinoma, rectum adenocarcinoma.; Mesothelioma: mesothelioma.; Ovarian: ovarian serous cystadenocarcinoma.; Biliary: pancreatic adenocarcinoma, cholangiocarcinoma.; Liver: liver hepatocellular carcinoma.; Testicular: testicular germ cell tumor.; Cervical: cervical & endocervical cancer.; Urinary: kidney papillary cell carcinoma, kidney clear cell carcinoma, bladder urothelial carcinoma, kidney chromophobe.; Adrenal: pheochromocytoma & paraganglioma, adrenocortical cancer.; Blood: acute myeloid leukemia, diffuse large B-cell lymphoma.; Melanoma: skin cutaneous melanoma, uveal melanoma.; Uterine: uterine corpus endometrioid carcinoma, uterine carcinosarcoma.; Prostate: prostate adenocarcinoma.; Thyroid: thymoma, thyroid carcinoma.

Genetic dependency data were from the Project Achilles CRISPR-Cas9 genome scale loss of function screening effort of cancer cell lines (release 21q4)^40–42^ (https://depmap.org/portal/download/). These data correspond to a 3-week fitness screen using the Avana sgRNA library. Chronos scores were used to quantify the fitness effect of individual gene loss, with “essentiality scores” in this paper represented as the Chronos score multiplied by −1. For example, a highly essential gene might have a Chronos fitness effect of −1.5 and thus an essentiality score of 1.5.

Processed cancer cell line gene expression and mutation data came from the Cancer Cell Line Encyclopedia (CCLE) and were obtained via download (21q4 release) from the DepMap portal (https://depmap.org/portal/download/). For analyses comparing cell lines by *HAPSTR1* mutation status, cells were considered to have at least one allele of a non-silent *HAPSTR1* mutation. We note that there are no hotspot mutations in *HAPSTR1* and these are thus likely to be loss-of-function mutations.

### Protein structure prediction

Structures were predicted using AlphaFold2, as implemented by ColabFold^2,10,11^. The settings were as follows, msa_mode: MMseqs2 (UniRef+Environmental), pair_mode: unpaired+paired, model_type: auto (AlphaFold2-ptm and AlphaFold-multimer v2), and num_recycles: 3. Resultant HAPSTR2 PDB files were aligned with HAPSTR1 structures weighting the 80-152 region (which is most confidently predicted in HAPSTR1 and comprises a functional HBO domain) using the PyMOL2 command “new_structure AND resi 80-152, reference_structure”.

### Cell lines

Cell lines were obtained from ATCC (HEK293T: CRL-3216, U2OS: HTB-96, H661: HTB-183) and cultured according to the manufacturer’s guidelines. Cells were passaged with Accumax and tested regularly for Mycoplasma contamination. Stable-engineered cell lines will be shared upon request to the corresponding author.

### *HAPSTR2* mutagenesis and gateway cloning

The human coding sequence for *HAPSTR2* (Ensembl) was synthesized (Twist Biosciences) with flanking attB fragments for Gateway BP cloning. From this template, HAPSTR2-G116R was cloned by overlap extension PCR. Mutagenic primers were designed (see Supplementary Table 1) to first amplify the region up- and downstream of the mutation. These products were gel purified (Qiagen) and then used as a template for a second PCR, which comprised 15 cycles without primers and then 30 cycles with primers flanking the start and stop codon. The ΔNLS (1-249) HAPSTR2 mutant was also cloned from this template using attB-flanked primers to achieve an amplicon comprising residues 1-253 and introducing a stop codon (see Supplementary Table 1). *HAPSTR2* constructs were then cloned into the Gateway donor vector pDONR221, and from there into expression vectors: pDest-HisMBP (Addgene 11085), pLenti6.2 3XFLAG-V5-ccdB (Addgene 87072), or pLenti CMV Hygro DEST (Addgene 17454). All PCRs used the 2x CloneAmp HiFi polymerase premix (Takara) and followed the manufacturer’s protocol for cycle number and length. All plasmids were verified through Sanger sequencing.

### Transfections and lentiviral infections

Smart-pool siRNAs (Horizon/Dharmacon) were obtained for each target gene of interest, as well as a non-targeting sequence, and transfected using RNAimax (Thermo Fisher) using the manufacturer’s recommended protocol (see key resource table for sequences of individual siRNAs in pool). Cells were harvested 72 h after siRNA transfection by default. For “chronic” knockdown experiments, cells were in siRNA for at least 24 h longer than necessary to achieve knockdown before performing the next phase of the experiment, at which point the siRNA transfection was repeated to prevent any loss of knockdown efficacy. “Acute” transfections indicates that cells were harvested 48 hours after introduction of nucleic acid. Knockdown was confirmed for each siRNA experiment by qPCR or immunoblot. Plasmid transfections used Lipofectamine 3000 (Thermo Fisher) and followed the manufacturer’s protocols.

For lentivirus, lentiviral vectors containing DNA constructs of interest were reverse co-transfected with pMD2.G and psPAX2 into 293T cells using Lipofectamine 3000 at the ratio recommended by the manufacturer. Media on transfected cells was changed 16 hours post-transfection, then the new virus-containing media was harvested 48 hours after initial transfection. Media was then centrifuged at 1000 × *g* and filtered with an 0.45 µm filter to yield packaged lentivirus. Lentivirus was then added directly to cells for 24 hours for transduction. Selection was achieved using 2 μg/ml puromycin, 10 μg/ml blasticidin, or 250–500 μg/ml hygromycin. Empty vector was used as a control for stable cell line experiments.

### Cell lysis and immunoblots

Unless otherwise specified, cells were lysed in buffer composed of: 50 mM Tris pH 7.5, 100 mM NaCl, 1% Triton, 0.2 mM EDTA, 5% Glycerol v/v, and 1 mM PSMF. Cell lysis was achieved by vortexing on ice followed by sonication using a Bioruptor (Diagnenode) for 10 cycles of 30 seconds on, 30 seconds off. Lysates were cleared by centrifugation for 10–15 min at 21,000 × *g* and 4 °C. NUPAGE 4-12% Bis-Tris gradient gels were used for all immunoblots (Thermo Fisher). Imaging was performed with the BioRad ChemiDoc Touch Imaging System (732BR0783) after incubation in HRP substrate (Immobilon, Millipore). Blots were analyzed using ImageLab v6.0.1 (BioRad). The antibodies used were as follows (name, catalog number, dilution). HAPSTRl Origene OTl2B8 (1:1000), FLAG Sigma F3165 (1:5000), HA Thermo 26183 (1:5000), HUWE1 Abcam ab70161 (1:1000), Vinculin Sigma V9131 (1:10,000), HO-1/HMOXl Novus NBPl-97507 (1:1000), p21/CDKN1A CST 2947 (1:1000), p53/TP53 Sigma P6749 (1:2000), HRP anti-rabbit IgG secondary CSF 7074 (1:10000), HRP anti-mouse IgG Thermo 31430 (1:10,000)

### Cycloheximide (CHX) chase experiments

293T cells stably expressing HAPSTR1-HA and HAPSTR2-FLAG were reverse transfected with non-targeting or HUWE1 siRNAs in 10 cm plates in triplicate, allowed to grow for 48 hours, then split and plated into 6-well plates at 7e5 cells per well in a 6-well plate. The subsequent morning, CHX (40 µg/mL) was added and plates were harvested either immediately (t_0_) or at every subsequent 2.25 hour timepoint. Protein abundance for each paralog was then quantified relative to t_0_ and an exponential decay curve was fit to the average of each replicate.

### Immunoprecipitations and amylose pulldown

Immunoprecipitations from mammalian cell lysates used anti-FLAG or anti-HA magnetic beads (Thermo Fisher), which were pre-washed 3× with lysis buffer (described above). Protein was then added to the beads before overnight incubation while rotating at 4 °C. Beads were washed 3× in lysis buffer prior to elution using glycine or being boiled in 2× SDS sample buffer.

Amylose pulldown experiments used 100 μl of amylose resin (New England BioLabs, E8021S), which was pre-washed 5x with wash buffer (10 mM Tris-HCl, 200 mM NaCl, 1 mM EDTA, 1 mM DTT, pH 7.5) before being incubated with either 750 μl of wash buffer, 750 μl of 5 μM purified His6-MBP, or His6-MBP-HAPSTR1 (rotating shaker, 2 hours at 4 °C). Control beads and amylose-bound recombinant protein were washed 5x using wash buffer. 2.5 mg of protein from 293T whole cell lysates was then added to protein-immobilized beads and incubated overnight at 4 °C. The protein captured mixture was washed 5× with wash buffer before being eluted using 150 μl of elution buffer (10 mM Tris-HCl, 200 mM NaCl, 1 mM EDTA, 1 mM DTT, 10 mM Maltose, pH 7.5) for 2 hours at 4 °C.

### Recombinant protein purification

HAPSTR1 protein was purified and characterized previously^2^. HAPSTR2 wild-type (WT) or G116R was cloned into pDest-HisMBP (Addgene 11085) as described above. Vectors were transformed into BL21 (DE3) cells and single clones were picked and sequence verified. Bacteria were then grown from glycerol stocks at 37 °C overnight in 5 mL liquid cultures containing ampicillin. Overnight cultures were inoculated into 1 L ampicillin-containing LB flasks and grown to an OD of 0.6–0.9 as measured by Nanodrop. Pre-induction bacteria was taken as a technical control and then IPTG was added to a concentration of 1 mM before shaking the flasks overnight at 16 °C. Cultures were pelleted at 4200 × *g* for 15 minutes at 4 °C before lysis in lysis buffer (20 mM Tris, 500 mM NaCl, 30 mM Imidazole, 0.25% CHAPS, pH 8.0, 0.5 U/mL Benzonase, 1 mg/mL Lysozyme, 1 mM PMSF, and Roche protease inhibitor cocktail). Cells were rotated end over end at 4 °C for 30 minutes then sonicated, 30 seconds on and 1 minute off, for five cycles at 4 °C. Lysates were then spun at 10,000 g for 30 minutes, the supernatants isolated, and spun again. Cleared supernatants were then applied to washed Ni-NTA agarose (Qiagen) in a 20 mL gravity column (BioRad). Bound proteins were washed four times with 10 mL of wash buffer (20 mM Tris, 500 mM NaCl, 30 mM Imidazole, 0.25% CHAPS, 5% glycerol, pH 8.0) prior to elution in 2–10 mL of elution buffer (20 mM Tris, 300 mM NaCl, 500 mM imidazole, 5% glycerol, pH 8.0). Proteins were then dialyzed (Pierce Slide-A-Lyzer Mini) to remove imidazole and concentrated using Amicon 10 kDa filters (EMD Millipore).

### Size exclusion chromatography

500 µg of each protein was run on a Superdex 200 Increase 10/300 GL pre-equilibrated with PBS containing 1 mM DTT using the ÄKTA pure chromatography system (GE Healthcare). A flow rate of 0.3 mL/minute was used for the column at 4 °C. Electrophoresis of eluted proteins and comparison with standard tracings facilitated estimates of complex stoichiometry at different elution volumes. Two batches of protein were tested to ensure the reliability of findings when comparing WT vs mutant chromatograms.

### Reverse-transcription quantitative real-time PCR

RNA was extracted using the RNeasy Mini Kit (Qiagen) including the optional QIAshredder and DNase treatment steps. Reverse transcription was achieved using the High Capacity cDNA Reverse-Transcription Kit (Applied Biosystems) using 1000 ng of input RNA, following manufacturer’s recommendations. Finally, qPCR was performed using primers specific to HAPSTR1, HAPSTR2, or ACTB (see below) using iTaq Sybr Green Supermix (BioRad). The cycling protocol was 95 °C × 3 minutes followed by 39 cycles of [95 °C × 10 seconds, 55 °C × 60 seconds]. No template controls (water) were free of significant amplification and gel electrophoresis of the qPCR confirmed a single product at the correct size.

### Immunofluorescence and microscopy

Cells were grown on poly-d-lysine treated sterile coverslips in a 24-well plate. Steps were performed at room temperature unless otherwise specified. Cells were washed three times with cold PBS, fixed with 4% paraformaldehyde for 10 min, and permeabilized with 0.2% Triton X100 for 5 min. Blocking encompassed incubation in 2% FBS for 30 min. Anti-FLAG (M2, Sigma) antibody was diluted at 1:250 and secondary antibodies were diluted at 1:1000 in 2% FBS and exposed to cells for 1 hour each, with 3 PBST washes between. Coverslips were mounted to a slide using a DAPI/mounting mixture (ProLong Antifade, Thermo Fisher) and allowed to dry overnight before imaging. Images were acquired using a Zeiss LSM800 confocal microscope using the Zen imaging system. A z-stack slicing distance of less than 0.9 µM was used. No non-linear adjustments were performed.

### Mass spectrometry

FLAG co-IPs from empty vector (EV), FLAG-HAPSTR1, or FLAG-HAPSTR2 293T cells were eluted from beads, run on a gel for 5 minutes, and submitted as a stacking gel band to the Northwestern University Proteomics Core Facility for an in-gel digestion. Peptides were analyzed by LC-MS/MS using a Dionex UltiMate 3000 Rapid Separation nanoLC coupled to a Orbitrap Elite Mass Spectrometer (Thermo Fisher Scientific Inc, San Jose, CA). Samples were loaded onto the trap column (150 μm × 3 cm inhouse packed with 3 μm ReproSil-Pur® beads). The analytical column was a 75 μm × 10.5 cm PicoChip column packed with 3 μm ReproSil-Pur® beads (New Objective, Inc. Woburn, MA). The flow rate was kept at 300 nL/min. Solvent A was 0.1% FA in water and Solvent B was 0.1% FA in ACN. The peptide was separated on a 120-min analytical gradient from 5% ACN/0.1% FA to 40% ACN/0.1% FA. MS1 scans were acquired from 400–2000 m/z at 60,000 resolving power and automatic gain control (AGC) set to 1 × 106. The 15 most abundant precursor ions in each MS1 scan were selected for fragmentation by collision-induced dissociation (CID) at 35% normalized collision energy in the ion trap. Previously selected ions were dynamically excluded from re-selection for 60 seconds. Proteins were identified from the MS raw files using Mascot search engine (Matrix Science, London, UK; version 2.5.1). MS/MS spectra were searched against the UniProt Human database (SwissProt 2022). Three missed tryptic cleavages were allowed. The MS1 precursor mass tolerance was set to 10 ppm and the MS2 tolerance was set to 0.6 Da. The search result was visualized by Scaffold (version 5.1.2, Proteome Software Inc., Portland, OR). Peptide identifications were accepted if they could be established at greater than 90.0% probability by the Peptide Prophet algorithm with Scaffold delta-mass correction. Protein identifications were accepted if they could be established at greater than 99.0% probability and contained at least 1 identified peptide. Protein probabilities were assigned by the Protein Prophet algorithm. Proteins that contained similar peptides and could not be differentiated based on MS/MS analysis alone were grouped to satisfy the principles of parsimony.

For our analyses, data in the Scaffold viewer were further filtered to a 1% false discovery rate for protein identification with a minimum of 2 peptides and 90% peptide threshold. Total spectra data were then exported and abundances were compared with the empty vector (EV) control IPs performed and analyzed in parallel. At least one sample needed >5 spectra to be included in our analysis. The average of two biological replicates is visualized for each condition.

### CRISPR-Cas9 knockouts

Individual sgRNAs were cloned into the LentiCRISPRv2 vector containing either blasticidin (Addgene 83480) or puromycin (Addgene 52961) selection cassettes (see Supplementary Table 1). To match the number of lentiviral infections and selection conditions, cells received the following pairs of sgRNA-containing vectors: WT (sgAAVS1 and sgNT), HAPSTR1-KO (sgHAPSTR1 and sgNT), HAPSTR2-KO (sgHAPSTR2 and sgNT), or DKO (sgHAPSTR1 and sgHAPSTR2). Note that AAVS1 served as a cutting control in the WT cells. Population knockouts were then created by high MOI lentiviral co-infection and dual antibiotic selection. The HAPSTR guides used for H661 stress assays were guides HAPSTR2-2 and HAPSTR1-2 (see Supplementary Table 1).

### Stress resilience assays

H661 cells were plated into 384-well plates at 500 cells per well in 50 µL of RPMI buffer supplemented with 10% FBS and 1% pen strep. Edge wells were not used and were instead filled with media. Immediately after addition to the plate, drugs or DMSO (vehicle) were dispensed to the desired concentration using a Tecan D300E drug printer. Cell counts over time were then assessed using the Incucyte live cell imaging apparatus. Five replicates were used per condition. Growth was quantified as the difference in the confluence between time 0 and time 120 and normalized to the difference for that genotype in untreated cells. To demonstrate effect size for normal cells and verify drug potency, doublings for WT cells are shown as a fold-change versus wells that received no drug nor vehicle control. To be included in figures, the WT cells had to increase in abundance (at least 3% absolute increase in confluence) over the time of the assay. The drug concentrations (µM) used were as follows: ATR inhibitor (ATRi) AZD6738: 0.128, 0.28, 0.6, 1.28, 2.8, 6.0; Camptothecin (CPT): 0.0248, 0.05, 0.096, 0.2; Colchicine (Colch): 0.0072, 0.0152, 0.0304; Neddylation inhibitor (NAEi) MLN4924: 0.104, 0.232, 0.496, 1.08, 2.32, 5.0.

### RNA-sequencing and gene set enrichment analysis

RNA was extracted using a Qiagen RNeasy kit following the manufacturer’s recommended protocol including the optional DNase treatment. Poly(A) mRNA was enriched using the NEBNext magnetic isolation module (E7490). Libraries were prepped using the NEBNext Ultra II Directional RNA Library Prep Kit (E7760), analyzed for quality using the Agilent High Sensitivity DNA kit, and quantified using the Qubit dsDNA HS assay. Libraries were pooled at 25 nM each, denatured with 1 M NaOM to a 0.2 M final concentration (5 min room temperature), and quenched with 200 mM Tris-HCl (pH 7). Pooled, denatured libraries were run on an Illumina NovaSeq using an SP Reagent kit (100 cycles) and paired-end read parameters. Analysis of RNA-sequencing data used the Ceto pipeline (https://github.com/ebartom/NGSbartom) with standard parameters, i.e. trimming with Trimmomatic 0.33, quality control with FastQC 0.11.2, alignment with STAR 2.5.2, counting with htseq 0.6.1, and normalization/differential expression analysis using EdgeR. Differential expression was considered at false discovery rate <5% with additional fold-change cutoffs when indicated. Gene Set Enrichment Analysis^43^ (GSEA) of differentially expressed genes (by cluster) was performed using the Molecular Signature DataBase as accessible at http://software.broadinstitute.org/gsea/msigdb/annotate.jsp. The gene sets queried were as follows: hallmark (H), KEGG pathways (C2), REACTOME (C2), GO Biological Process (C5), and GO Molecular Function (C5).

### Statistics and data analysis

All data analysis was performed using standard modules in Python (v3.7.6) as follows. Bar, box, line, strip (individual point), and violin plots were created using the respective functions in Seaborn (v0.11.1) and Matplotlib (v3.5.2). Data cleaning and statistical analyses used standard functions in Pandas (v1.1.3), Numpy (v1.21.1), Scipy (v1.6.2), and Statannot (v.0.2.3). Individual replicates highlighted in figures and figure legends refer to biological replicates (independent samples/experiments) rather than technical replicates. Where technical replicates were performed, the values were averaged to a single biological replicate value.

### Reporting summary

Further information on research design is available in the Nature Portfolio Reporting Summary linked to this article.

## Supplementary information

Supplementary Information

Description of Additional Supplementary Files

Supplementary Data 1

Supplementary Data 2

Supplementary Data 3

Supplementary Data 4

Reporting Summary

## Data Availability

All data generated during this study are included in this published article and its supplementary information files. Original source data for analyses using public databases were obtained from GTEx v8 (https://GTExportal.org/home/), ENCODE (https://www.encodeproject.org/), TCGA via UCSC XenaBrowser (https://xenabrowser.net/datapages/), DepMap/CCLE (21q4 release, depmap.org/portal/download/), or MGI (informatics.jax.org) as described in Methods. Data to reproduce figures and uncropped immunoblot images for are available in the Source Data file. The RNA-sequencing data generated in this study have been deposited in the GEO database under accession code GSE219209. The proteomic data generated in this study have been deposited in the PRIDE database under accession code PXD038642. Source data are provided with this paper.

## Source data

Source Data
