# Supplementary material for: The HAPSTR2 retrogene buffers stress signaling and resilience in mammals
Source: Nat Commun. 2023 Jan 11;14:152. doi: 10.1038/s41467-022-35697-1 (PMC9834230; doi:10.1038/s41467-022-35697-1)
Supplement: Supplementary file 1 — Supplementary Information [file 41467_2022_35697_MOESM1_ESM.pdf]

Supplementary Information accompanying:

The HAPSTR2 retrogene buffers stress signaling and resilience in mammals

by D.R. Amici et al.

Nature Communications

Supplementary Figure 1: Further analysis of the HAPSTR2 genomic DNA sequence. Retrocopies insert into the genome with their ancestor's 5' untranslated region (UTR), typically including a poly(A) addition signal and a poly(A) tail. The LINE-1 retrotransposon machinery also leaves a target site duplication scar flanking this tail and the 3' sequence. While these sequences represent evidence for a retrocopy's origins as a mature mRNA, they are expected to degenerate substantially over time due to lack of selection[1]. Here, we identify remnants in HAPSTR2 which have characteristics of these retrogene signatures. Note key at bottom of figure. Numbers indicate nucleotide position relative to beginning of start codon.

-1148 TCTCGTGTTTGA -1137
-495 TCCTGCC -489

1 ATG GAG GAG CAG CAG AAG GAG GGC GAG GCC GAG GTC GCG GAG CAC TGG TTT TCC AAG TGG GAG CGC CAG TGC CTG GCT GAG GCC GAG CAG GAG 94
1 Met Glu Glu Gln Gln Lys Glu Gly Gly Ala Glu Val Ala Glu His Trp Phe Ser Lys Trp Glu Arg Gln Cys Leu Ala Glu Ala Glu Gln Glu 31

93 GAG CAG CTG CCC CCC GAG CTG CAG GAG GAG GCG GCT GCA GAG TTG GCA GGG CTC AAG AGC GAG AAG CAG AAG CTG TGG CAC CTC TTC CAG ATC 186
32 Glu Gln Leu Pro Pro Glu Leu Gln Glu Glu Ala Ala Ala Glu Leu Ala Gly Leu Lys Ser Glu Lys Gln Lys Leu Trp His Leu Phe Gln Ile 62
↓↓
187 TCG GCC ACC GCC GTT GCT CAG CTT TAC AAG GAT TCT GGG TGC CAA CAG CAA GGA CTT TCC ATG TGG GAC CCC TTC CAG AAT GCG GCC ATG GCC 279
63 Ser Ala Thr Ala Val Ala Gln Leu Tyr Lys Asp Ser Gly Cys Gln Gln Gln Gly Leu Ser Met Trp Asp Pro Phe Gln Asn Ala Ala Met Ala 93
↓↓
280 GTG ACC AGC CTC TAC AAA GAG AGC GGG GAT GCC CAC CAA CGA AGT TTT GAC TTG GGT GTC CAG GTT GGC CAC CAG CGT CGC ATC AAA GAT GTG 372
94 Val Thr Ser Leu Tyr Lys Glu Ser Gly Asp Ala His Gln Arg Ser Phe Asp Leu Gly Val Gln Val Gly His Gln Arg Arg Ile Lys Asp Val 124

373 CTG GAG TGG GTG AAA AAG GGC CGG AGC ACC ATT CGT CGC GAA GAC TTG ATT AGC TTC CTG TGT GGC AAA GTG CCC CCC GCT CCT CCT CCA CCT 465
125 Leu Glu Trp Val Lys Lys Gly Arg Ser Thr Ile Arg Arg Glu Asp Leu Ile Ser Phe Leu Cys Gly Lys Val Pro Pro Ala Pro Pro Pro Pro 155

466 CGC ACT CCT AGG ACA CCC CCG AAG CCA CCC ACT GGG GTC ACC AGC CAG GCT GTG GCA ACT GAG TCC AGC TCA TCG GTG GAC GTC GAC CTG CAG 558
156 Arg Thr Pro Arg Thr Pro Pro Lys Pro Pro Thr Gly Val Thr Ser Gln Ala Val Ala Thr Glu Ser Ser Ser Ser Val Asp Val Asp Ley Gln 186
↓↓
559 CCC TTC CAG GAG GCG ATC GCC CTG CAT GGC CTC AGT GGT GCT ATG GCC GGC ATC AGC ATG CGA TCG GGC GAC TCG CCT CAA GAC AGC GGT GTC 651
187 Pro Phe Gln Glu Ala Ile Ala Leu His Gly Leu Ser Gly Ala Met Ala Gly Ile Ser Met Arg Ser Gly Asp Ser Pro Gln Asp Ser Gly Val 217

652 GCC AGC AGT GGG CGC CGA AAA ACT AGC TTC TTG GAG GAC GAC TTG AAT CCC TTC GAC TCA GAG GAA CTG GCC CTC CAC CTG GAC AGT GGG GGG 744
216 Ala Ser Ser Gly Arg Arg Lys Thr Ser Phe Leu Gly Asp Asp Ley Asn Pro Phe Asp Ser Gly Gly Ley Ala Ley His Ley Asp Ser Gly Gly 248

745 ATC CGC AAG CGC ACC TCG GCC AAT GCA GTG ATG GCA TCA CAG ACT CCC CAA TCC AAA AGC GCA AAC CGA ATG GTC TAA 822
*247 Ile Arg Lys Arg Thr Ser Ala Gln Cys Ser Asp Gly Ile Thr Asp Ser Pro Ile Gln Lys Arg Asn Arg Met Val * 273*

823 CTGCCTCATTGGTTGCCTGCCGCCATATTGCTTGAGAGTGAACTCAACCGTCGACATGCTTGTCTAAAGGTTACTGGAGACCATTTTTCTTCCCTTCTCTAAGTTAAACAAAGATTTCTAAC 945
946 ATTTTGCCATAAAGAAACTTTAAAAGTATTCCAGAAGAGGCTTCATATGACTCTGACTTTCCAAAAAATATAATCCTAGCAGAGAACAAATATGTAGTAGTTAGCAGGATCATTTAAAGCAA 1068
1069 CGTATCTGGTCAAGGCAGGAGTCTGATTTATCTTGTTCACAGTTATATTCCTCAGCACCTAGCACAGTTCCAGGTGCTCAATAAATGTTCATTGAATGAATAAATGTGACCTTATTTATTCT 1191
1192 AAAGGGAAGTCATAGCAGGTGTTTACTTGGTTATAGAATACTTTTTTCTTTGATAATTGTTGGCTTTAATGGTCTTTGCCAATTCAATTGCATTGTATCAAAATGTAAAACTTGACATCTTT 1314
1315 TGAAATCTGGAGTCTTCCATTTTTACCTCACTATACCATGCACCTAATTTCTAGAAAGAAACTGCAAGTAATACATATTAGGCTGTGATTTTTTTAATTTTGAAAAATTGTTGATTTTGGTG 1437
1438 TTATGTGATGCTGCAAAGAGGTGCTGTGGTGGTTACGCAGGTATTACTTAATATTGAGTCAATTCAGTGCTTTTTTGGGGAAAACTAAACCATCAACTGAGACCCTGATGAGATGGTGGCCC 1560
1561 GTGGTGCTCTTTTTTTTTAGGAATGTAAAAATTCCTTTTTCTGAGGTTACATCTTTTTTATGATCTCTGTTTTGAAGTGGAAGACCAAGTAGTCAGAATGAACTTCTGGGTTTTTTTTTTTT 1683
1684 TTCTTTTTTTTTTTTTTTTAAGACAGAGCCTCGCACTGTTGCCCGGGCTGGAGTGCAATGGCGTGATCTCGGCTCACTGCAACCTCCGCCTCCCGGGTTCAAGGGATTCTCCTGCCTCAGCC 1806
1807 CCCGAGAAGCTGGGATTGCAGGTGCCTGCCACCACGCCCGGCTAATTTTTTGTGTTTTTAGTAGAGACGGGGTTTCACTATGTTGGCCAGGCTGGTCTCGAACTCCTGACCTTGTGATCCGC 1929
1930 CACCTTGGCCTCCCAAAGTGCTGGGATTACAGGCGTGAGCCACCGTGCCCAGCCTTTTGAACTTCTGGGTTTTAATTAAGGGTACAGCATGGTATAACCAAAAGAGTGTGAACTTCGTTTTA 2052
2053 AAGGACCAACAGCAGAATCCTAGCTCTATAAATTAGCTGCATGATTTTGAACAAGTTACTTAATGTCTATTGACCTCAGTTTTTCATCTGTAAAATGGAACTGTGTGTCTCCTAAGGTGGTT 2175
2176 TGAGGATCAGGTGAAATTTATATTTCAAGTGTCCAGCACAATCTTGGCACATAAAATACTC**AATAAA**GGTTCTCCCTTCTCTATAAATAAAAATCAATAAAACATTAAAATCATTTTATTTT 2298
2299 GAAAAAGCAGAAAAGTAGATCAGCTTTAAAATCAGCAAGTAGAATCTCTGTGTTTGAACTGGAAATTACTGTTTCACATTAGAAACTTGCCTAAATATCACAGATCTTGTGTAAGTGTGTTG 2421
2422 GCTCATGAGCACTCAGGGTCACCAATCTGGATACTAGGGCAACCTTGGAGTTCCTGAAGTTGCCTGAAGGATGCACTTTGCAATTAGGGGTAATGAAACATCTACCACCTCTGGCCTGAAAA 2544
2545 ATATGAGGAGGAGGGTGTAAAGTAGAAAAAAATTGGAAGCCAAGGTCCTAGGGCACCTAAAAGGCTAAAGTGGTTTTAAATGAGGTTGGGGTAGGGTGGGGTGGGGTGGTGTGGAGGCGATG 2667
2668 AGGAGGTAGAGCAGGTGGGGATCTGGGGGGTTGGGGAGGGGAGGGGAGGTGGGAGTATGATTATTGAAGTGGGGGTGGGGAGTTAAAAAGCCCAATCTTCCCAGGGTCTCCCAAAACCAGTG 2790
2791 ATAAAATATTATAGATTGGCAAAACCTTGCCTTTATGTTCACACCAGAGTTGTTTCTGCTTCATTAGCATGGCCTTTCGAGTAGATTTGTAATTGATGAATGCATTAAAATAATAAAATAAT 2913
2914 CAAATTCCATGATATAATAAAAACTGGCTGGCCGGGCACGGTGGCTCATGCCTGTAATCCCAGCACTTTGGGAGGCCGAGGTGGGCAGATCACCTGAGGTCAGGAGTTCGAGACCAGCCTGG 3036
3037 CAACATGGTGAATGAAACCCCATCTCTACTAAAAATATAAAAATTAGCGGGGCGTGGTGGTGGGCACCTGTAATCCCAGCTACTCAGGAAGCTGAGGCAGGAGAATCACTTGAACCCAGGAG 3159
3160 TGGAGGTTGCAGTGAGCTGAGACCGCACCATTGCACTCCAGCCTGGGCGACAAGAGCAAAACTCCGTCTCAAAAAAAAAAAAAAAAAAAAAAAAAAAAAAAGAGAAAAAAACCCAGAGAAGAAC 3282
3283 GGCTATTGAGGTACTGTGCTAGGTCCTGAGAAGGGATATAAAAAAGCATAAAAACAATTCTTATCCTCAAAGAGCACATCCTGCCTTGGAAGATAAATTATGTAGCCAAAAGCACGCTTCTA 3405
3406 CTGGGAAAAGTGCCCAAGGGCTGTGCAGAATTCAGAGGTCAGAGACTCTCCTTGCTGAGAGGGTCACAGCTTCTGGAAGAAGTAGCATTTGAACCGGGCCCAGAAAAATGGGTAGGAAGGGA 3528

KEY
Changed nucleotides: X | ↓↓ Intron site in HAPSTR1 missing in HAPSTR2
Candidate retrogene signatures: target site duplication, poly(A) tail, and **nearby poly(A) addition signal**

NOTE
Candidate poly(A) 1 (2259-2343), 54.1% A; Candidate poly(A) 2 (3232-3361), 55.4% A

Supplementary Figure 2: Further analysis of HAPSTR gene and protein structures. a) Sequence and domains of HAPSTR proteins, as in Fig. 1E, but highlighting the degree to which individual residues have remained identical for each protein over their evolutionary lifespan. Note that HAPSTR2 (H2) covers much less evolutionary time than does HAPSTR1 (H1) and thus comparisons should be primarily intra-protein. Similarity was determined using the BLOSUM62 substitution matrix. **b)** AlphaFold2-derived prediction of HAPSTR2 protein structure aligned and compared with that of HAPSTR1. Highlighted is the largest discrepancy. A segment ending in HAPSTR1's proline 52, a glycine in HAPSTR2, promotes a second kink in the alpha helix that is predicted to be absent in HAPSTR2. N- and C- terminal amino acids (disordered, not confidently predicted) are hidden for visibility. **c-d)** Structure of a HAPSTR1 chromosomal gene segment duplication in fish (c) vs. a HAPSTR1 retrocopy insertion in mammals (d), with representative species shown. UTR, untranslated region; kb, kilobases.

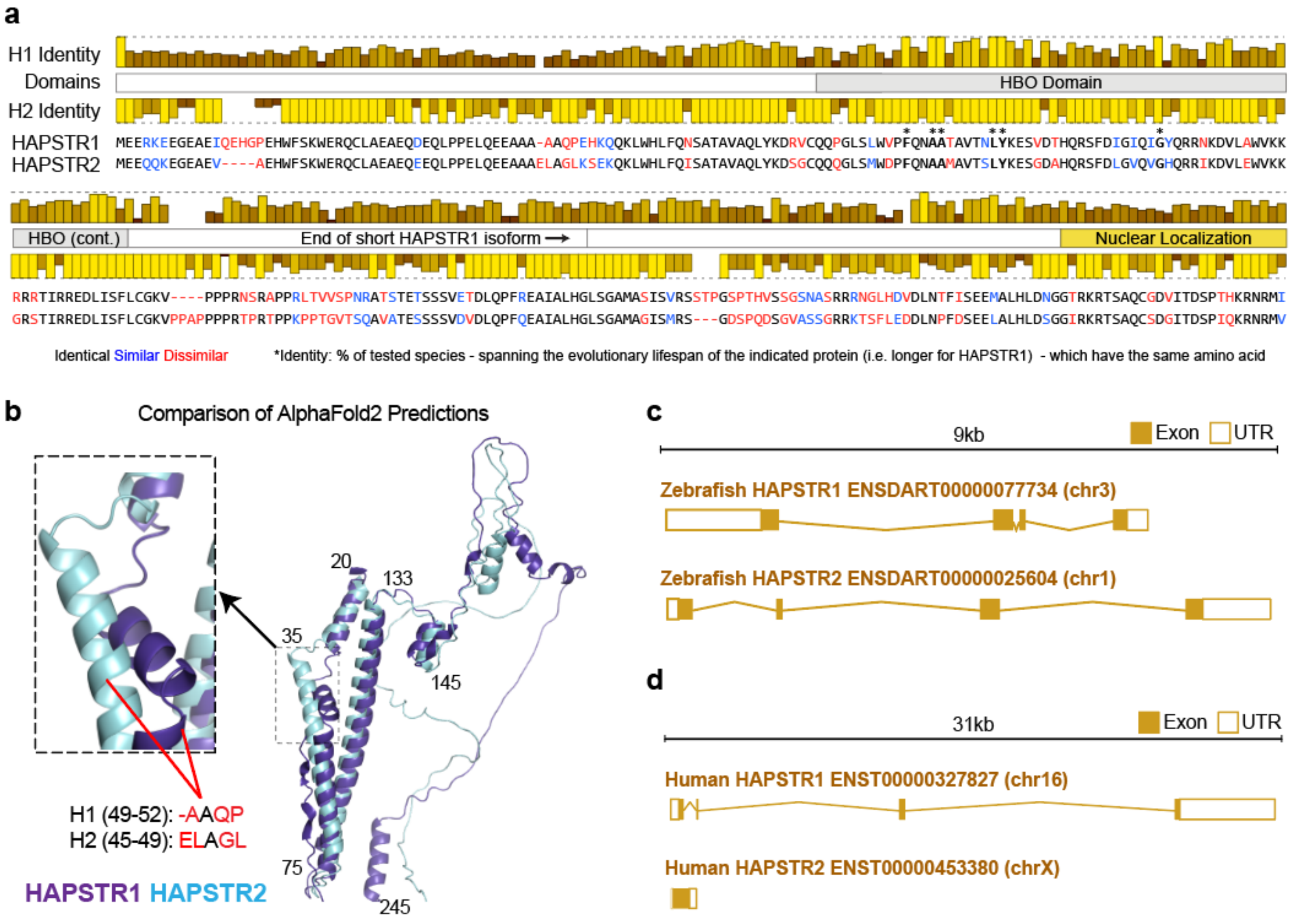

Supplementary Figure 3: Further analysis of HAPSTR2 transcription. a) RT-qPCR signal for HAPSTR2, relative to beta-actin, in three cell lines (293T, OVCAR3, H661) is proportional to the abundance estimates for HAPSTR2 in RNA-seq experiments. nd, no data for cell line. N=3 biologically independent samples shown. Mean +/- 95% confidence interval (CI). **b-c)** HAPSTR2 overexpression and knockdown validate primer specificity for HAPSTR2 qPCR (b) and indicate that HAPSTR2 siRNA (siHAPSTR2) does not impact HAPSTR1 transcript abundance (c). N=3 biologically independent samples shown**.** Mean +/- 95% CI. **d)** HAPSTR2 mRNA abundance relative to HAPSTR1 in human tumors from TCGA, i.e. HAPSTR2/HAPSTR1 * 100. Box is median and lower/upper quartile with whiskers double the interquartile range. N=10535. **e)** Pattern of HAPSTR2 gene expression in cancer cell line RNA-seq experiments; CCLE data. N=1825. Box is median and lower/upper quartile with whiskers double the interquartile range. **f-g)** HAPSTR2 expression is not induced in contexts of HAPSTR1 non-silent mutation and does not correlate strongly with HAPSTR1 expression levels or copy number; CCLE data. Two-tailed t-test (f). **h)** HAPSTR2's promoter region does not strongly align with that of HAPSTR1 as compared with an unrelated control gene. TPM, transcripts per million; L2, log_2; cDNA, complementary DNA.

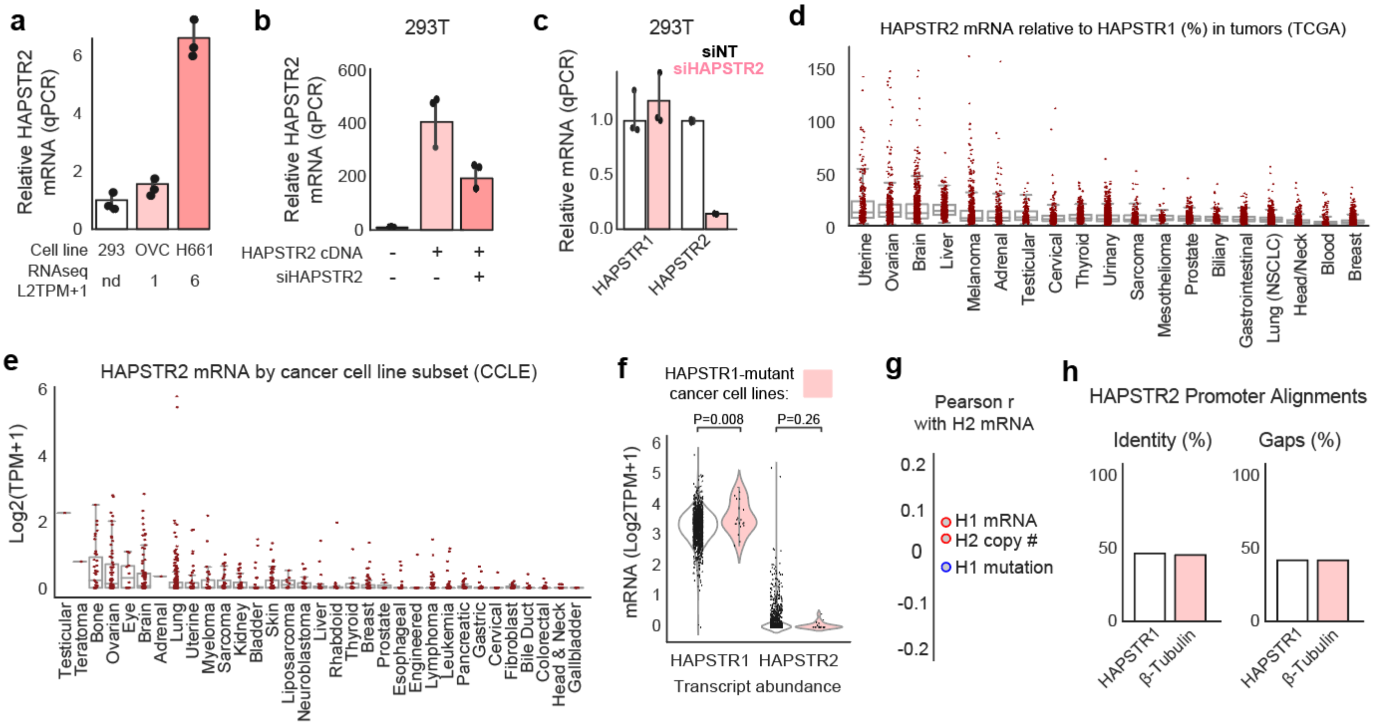

Supplementary Figure 4: Further analysis of the HAPSTR2 protein. a) FLAG (F)-HAPSTR2 transfection in 293T cells results in expression at the predicted size. Representative of N>10. EV, empty vector. **b)** Representative Coomassie-stained gel of 1 µg recombinant MBP-HAPSTR2. Like MBP-HAPSTR1, C-terminal truncation or cleavage fragments are present[2]. Representative of N=3. **c-d)** HAPSTR1 Clone 2B8 antibody (Origene) readily recognizes HAPSTR1 but less avidly recognizes HAPSTR2. 2-fold increase in lysate loaded in (d). Both representative of N=3. **e)** Representative immunoblot for stability experiments quantified in Fig. 3i, N=3. HAPSTR1 (H1), HAPSTR2 (H2), non-targeting (NT), hours (h), cycloheximide (CHX). **f)** Representative immunoblots for experiments quantified in Fig. 4d. N=3. Note that HAPSTR1 on immunoblot appears as a long (L) and short (S) isoform with a nonspecific (ns) band in between[2].

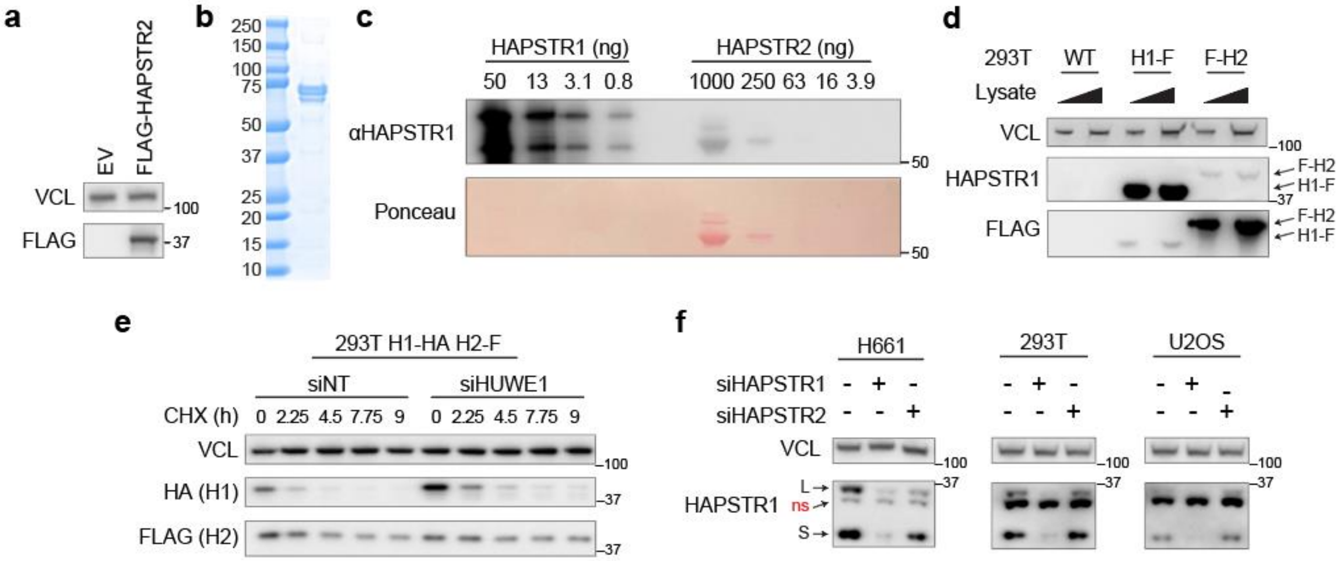

Supplementary Figure 5: Further analysis of HAPSTR depletion and knockdown genetic interaction experiments. a) Individual gene expression profiles for each gene in the indicated cluster, related to Fig. 5c. **b)** Confirmation of efficient knockdown and specificity of reagents in our H661 siRNA RNA-sequencing experiment. N=3 biologically independent samples. Mean +/- 95% confidence interval (CI). **c)** Schematic illustration of how different genetic interactions may be observed by comparing single and double loss-of-function experiments. **d)** Effect of CRISPR-Cas9 sgRNAs targeting HAPSTR1 (H1) or HAPSTR2 (H2) in HAPSTR2-expressing H661 cells and HAPSTR2-non-expressing 293T cells. N=3. Mean +/- 95% CI. Two-tailed t-test vs. sgAAVS1.

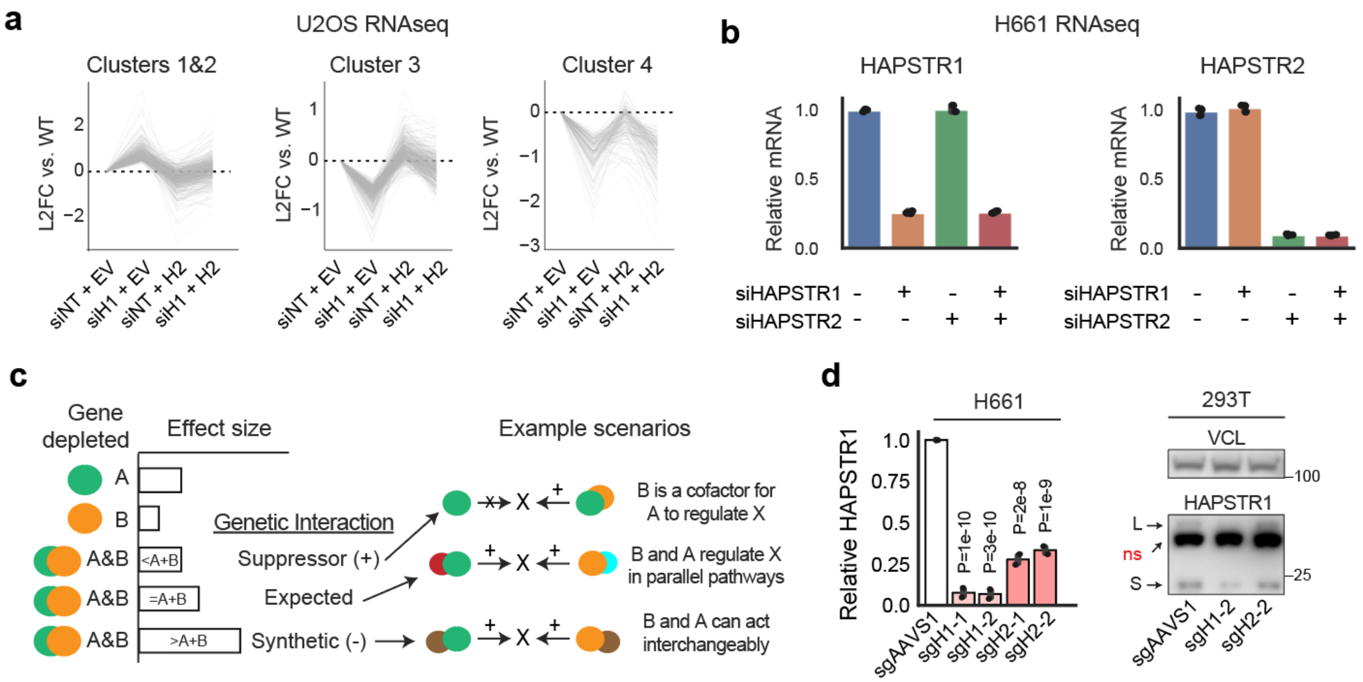

Supplementary Table 1

**HAPSTR2 gateway cloning and mutagenesis primers**	
hap2_attB_1F	ggggacaagtttgtacaaaaaagcaggcttaATGGAGGAGCAGCAGAAG
hap2_attB_273R	ggggaccactttgtacaagaaagctgggttTTAGACCATTCGGTTGCG
hap2_attB_249R	ggggaccactttgtacaagaaagctgggtttcaGATCCCCCCACTGTCCAGG
hap2_G116R_fwd	CCAGGTTCGCCACCAGCGTCGCATCAAAG
hap2_G116R_rev	TGGTGGCGAACCTGGACACCCAAGTC
**Real-time quantitative PCR primers**	
hsHAPSTR1_F	GTTCCTCCACCACGAAACTCT
hsHAPSTR1_R	TGCACCACTAAGACCATGCAGA
hsHAPSTR2_F	TGGAGGACGACTTGAATCCC
hsHAPSTR2_R	TTAGACCATTCGGTTGCGCT
hsACTB-F	CATGTACGTTGCTATCCAGGC
hsACTB-R	CTCCTTAATGTCACGCACGAT
**sgRNA sequences**	
AAVS1	GGGGCCACTAGGGACAGGAT
Non-Targeting	ATCGTTTCCGCTTAACGGCG
HAPSTR1-1	TTTGTAGAGATTGGTGACGG
HAPSTR1-2	GCAATAAGGATGTGTTGGCT
HAPSTR2-1	CATCTTTGATGCGACGCTGG
HAPSTR2-2	CAAGTCAAAACTTCGTTGGT
**Key antibodies**	
HAPSTR1	Origene OTI2B8
FLAG	Sigma F3165
HA	Thermo 26183
HUWE1	Abcam ab70161
Vinculin	Sigma V9131
HO-1/HMOX1	Novus NBP1-97507
p21/CDKN1A	CST 2947
p53/TP53	Sigma P6749
**siRNA sequences**	
HAPSTR2-1	GCGAAGACUUGAUUAGCUU
HAPSTR2-2	GCUCAGCUUUACAAGGAUU
HAPSTR2-3	GCUCAUCGGUGGACGUCGA
HAPSTR2-4	GCUUCUUGGAGGACGACUU
HAPSTR1-1	CUACAAAGACCGAGUGUGU
HAPSTR1-2	GAAUUCAGAUUGGCUAUCA
HAPSTR1-3	CAGAAGAACUAUUCGUCGA
HAPSTR1-4	GAUCUAAACUGCAAACAUU

Supplementary References

1 Kaessmann, H., Vinckenbosch, N. & Long, M. RNA-based gene duplication: mechanistic and evolutionary insights. *Nat Rev Genet* **10**, 19-31, doi:10.1038/nrg2487 (2009).

2 Amici, D. R. *et al.* C16orf72/HAPSTR1 is a molecular rheostat in an integrated network of stress response pathways. *Proc Natl Acad Sci U S A* **119**, e2111262119, doi:10.1073/pnas.2111262119 (2022).
