# Supplementary material for: The HAPSTR2 retrogene buffers stress signaling and resilience in mammals
Source: Nat Commun. 2023 Jan 11;14:152. doi: 10.1038/s41467-022-35697-1 (PMC9834230; doi:10.1038/s41467-022-35697-1)
Supplement: Supplementary file 2 — Description of Additional Supplementary Files [file 41467_2022_35697_MOESM2_ESM.pdf]

Description of Additional Supplementary Files:

The HAPSTR2 retrogene buffers stress signaling and resilience in mammals

by D.R. Amici et al.

Nature Communications

File Name: Supplementary Data 1
Description: Data from mass spectrometry analysis of HAPSTR1 and HAPSTR2 immunoprecipitations.

File Name: Supplementary Data 2
Description: Processed RNA-sequencing data from U2OS experiments.

File Name: Supplementary Data 3
Description: Processed RNA-sequencing data from H661 experiments.

File Name: Supplementary Data 4
Description: Results from queries of clinical variant databases for the HAPSTR2 gene.
