# Supplementary material for: The HAPSTR2 retrogene buffers stress signaling and resilience in mammals
Source: Nat Commun. 2023 Jan 11;14:152. doi: 10.1038/s41467-022-35697-1 (PMC9834230; doi:10.1038/s41467-022-35697-1)
Supplement: Supplementary file 7 — Reporting Summary [file 41467_2022_35697_MOESM7_ESM.pdf]

nature portfolio

Corresponding author(s): Marc Mendillo

Last updated by author(s): Dec 2, 2022

Reporting Summary

Nature Portfolio wishes to improve the reproducibility of the work that we publish. This form provides structure for consistency and transparency in reporting. For further information on Nature Portfolio policies, see our Editorial Policies and the Editorial Policy Checklist.

Statistics

For all statistical analyses, confirm that the following items are present in the figure legend, table legend, main text, or Methods section.

n/a	Confirmed	
☐	☒	The exact sample size (*n*) for each experimental group/condition, given as a discrete number and unit of measurement
☐	☒	A statement on whether measurements were taken from distinct samples or whether the same sample was measured repeatedly
☐	☒	The statistical test(s) used AND whether they are one- or two-sided<br>*Only common tests should be described solely by name; describe more complex techniques in the Methods section.*
☒	☐	A description of all covariates tested
☐	☒	A description of any assumptions or corrections, such as tests of normality and adjustment for multiple comparisons
☐	☒	A full description of the statistical parameters including central tendency (e.g. means) or other basic estimates (e.g. regression coefficient) AND variation (e.g. standard deviation) or associated estimates of uncertainty (e.g. confidence intervals)
☐	☒	For null hypothesis testing, the test statistic (e.g. *F*, *t*, *r*) with confidence intervals, effect sizes, degrees of freedom and *P* value noted<br>*Give P values as exact values whenever suitable.*
☒	☐	For Bayesian analysis, information on the choice of priors and Markov chain Monte Carlo settings
☒	☐	For hierarchical and complex designs, identification of the appropriate level for tests and full reporting of outcomes
☒	☐	Estimates of effect sizes (e.g. Cohen's *d*, Pearson's *r*), indicating how they were calculated

Our web collection on statistics for biologists contains articles on many of the points above.

Software and code

Policy information about availability of computer code

Data collection	RNA-sequencing used an Illumina Novaseq 6000. Quantitative PCR used a Bio-Rad CFX96 Real-Time System. Microscopy images were obtained using an LSM800 inverted confocal using the Zen system. Immunoblots were imaged using the Bio-Rad ChemiDoc Touch Imaging System (732BR0783). Mass spectrometry used a Dionex UltiMate 3000 Rapid Separation nanoLC coupled to a Orbitrap Elite Mass Spectrometer and the Mascot search engine. Sequence alignments used Clustal Omega v1.2.4 as accessed at https://www.ebi.ac.uk/Tools/msa/clustalo/, with visualization of the alignment in Jalview v2.11, or Snapgene (v5.3) where indicated.
Data analysis	All data analysis was performed using standard modules in Python (v3.7.6) as follows. Bar, box, line, strip (individual point), and violin plots were created using the respective functions in Seaborn (v0.11.1) and Matplotlib (v3.5.2). Data cleaning and statistical analyses used standard functions in Pandas (v1.1.3), Numpy (v1.21.1), Scipy (v1.6.2), and Statannot (v.0.2.3). RNA-sequencing analysis used the Ceto pipeline (github.com/ebartom/NGSbartom). Blots were analyzed using ImageLab v6.0.1 (BioRad).

For manuscripts utilizing custom algorithms or software that are central to the research but not yet described in published literature, software must be made available to editors and reviewers. We strongly encourage code deposition in a community repository (e.g. GitHub). See the Nature Portfolio guidelines for submitting code & software for further information.

Data

Policy information about availability of data

All manuscripts must include a data availability statement. This statement should provide the following information, where applicable:

- Accession codes, unique identifiers, or web links for publicly available datasets
- A description of any restrictions on data availability
- For clinical datasets or third party data, please ensure that the statement adheres to our policy

All data generated during this study are included in this published article and its supplementary information files. Original source data for analyses using public databases were obtained from GTEx v8 (https://GTExportal.org/home/), ENCODE (https://www.encodeproject.org/), TCGA via UCSC XenaBrowser (https://xenabrowser.net/datapages/), DepMap/CCLE (21q4 release, depmap.org/portal/download/), or MGI (informatics.jax.org) as described in Methods. Data to reproduce figures and uncropped immunoblot images for are available in the Source Data file. The RNA-sequencing data generated in this study have been deposited in the GEO database under accession code GSE219209 (https://www.ncbi.nlm.nih.gov/geo/query/acc.cgi?acc=GSE219209). The proteomic data generated in this study have been deposited in the PRIDE database under accession code PXD038642 (https://www.ebi.ac.uk/pride/archive/projects/PXD038642)

Human research participants

Policy information about studies involving human research participants and Sex and Gender in Research.

Reporting on sex and gender	N/A
Population characteristics	N/A
Recruitment	N/A
Ethics oversight	N/A

Note that full information on the approval of the study protocol must also be provided in the manuscript.

Field-specific reporting

Please select the one below that is the best fit for your research. If you are not sure, read the appropriate sections before making your selection.

☒ Life sciences ☐ Behavioural & social sciences ☐ Ecological, evolutionary & environmental sciences

For a reference copy of the document with all sections, see nature.com/documents/nr-reporting-summary-flat.pdf

Life sciences study design

All studies must disclose on these points even when the disclosure is negative.

Sample size	3-5 samples were chosen per group based on the degree of variance observed for prior similar experiments in the lab. Refer to figure legends for exact sample sizes.
Data exclusions	No data exclusions
Replication	All experiments were repeated at least 3x as indicated in the manuscript.
Randomization	N/A
Blinding	No blinding

Reporting for specific materials, systems and methods

We require information from authors about some types of materials, experimental systems and methods used in many studies. Here, indicate whether each material, system or method listed is relevant to your study. If you are not sure if a list item applies to your research, read the appropriate section before selecting a response.

Materials & experimental systems

n/a	Involved in the study
☐	☒ Antibodies
☐	☒ Eukaryotic cell lines
☒	☐ Palaeontology and archaeology
☒	☐ Animals and other organisms
☐	☒ Clinical data
☒	☐ Dual use research of concern

Methods

n/a	Involved in the study
☒	☐ ChIP-seq
☒	☐ Flow cytometry
☒	☐ MRI-based neuroimaging

Antibodies

Antibodies used

HAPSTRl Origene OTl2B8 (1:1000)
FLAG Sigma F3165 (1:5000)
HA Thermo 26183 (1:5000)
HUWE1 Abccam ab70161 (1:1000)
Vinculin Sigma V9131 (1:10000)
HO-1/HMOXl Novus NBPl-97507 (1:1000)
p21/CDKN1A CST 2947 (1:1000)
p53/TP53 Sigma P6749 (1:2000)
HRP anti-rabbit IgG secondary CSF 7074 (1:10000)
HRP anti-mouse IgG Thermo 31430 (1:10000)

Validation

All verified by comparison with molecular weight marker and positioning on gel. Knockout/knockdown/overexpression studies were used for HAPSTRl/HA/FLAG/HUWEl. Commercial validation for other antibodies can be found:
Vinculin (specific 116 kDa band, https://www.sigmaaldrich.com/US/en/product/sigma/v9131)
HO-1 (specific 33 kDa band https://www.novusbio.com/products/ho-1-hmox1-hsp32-antibody-ho-1-1_nbp1-97507)
p21 (KO verified https://www.cellsignal.com/products/primary-antibodies/p21-waf1-cip1-12d1-rabbit-mab/2947)
p53 (specific 53 kDa band https://www.sigmaaldrich.com/US/en/product/sigma/p6749)
Rabbit secondary (thoroughly referenced https://www.cellsignal.com/products/secondary-antibodies/anti-rabbit-igg-hrp-linked-antibody/7074)
Mouse secondary (thoroughly referenced https://www.thermofisher.com/antibody/product/Goat-anti-Mouse-IgG-H-L-Secondary-Antibody-Polyclonal/31430)

Eukaryotic cell lines

Policy information about cell lines and Sex and Gender in Research

Cell line source(s)	ATCC. HEK293T: CRL-3216, U2OS: HTB-96, H661: HTB-183
Authentication	All cell lines derived from early passages of an authenticated ATCC stock
Mycoplasma contamination	Regular testing, always negative
Commonly misidentified lines (See ICLAC register)	None

Clinical data

Policy information about clinical studies
All manuscripts should comply with the ICMJE guidelines for publication of clinical research and a completed CONSORT checklist must be included with all submissions.

Clinical trial registration	N/A
Study protocol	N/A
Data collection	N/A
Outcomes	N/A
